# Supplementary material for: Transcriptome profiling of type VI secretion system core gene tssM mutant of Xanthomonas perforans highlights regulators controlling diverse functions ranging from virulence to metabolism
Source: Microbiol Spectr. 2023 Nov 29;12(1):e02852-23. doi: 10.1128/spectrum.02852-23 (PMC10782981; doi:10.1128/spectrum.02852-23)
Supplement: Supplemental material — Fig. S1 and Tables S1 to S13. [file spectrum.02852-23-s0001.pdf]

A.

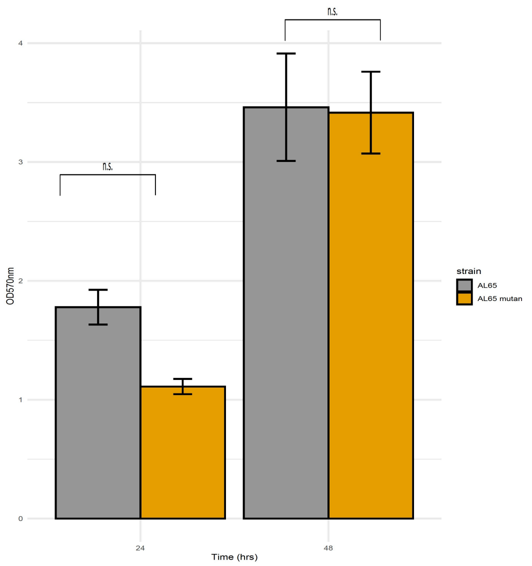

B.

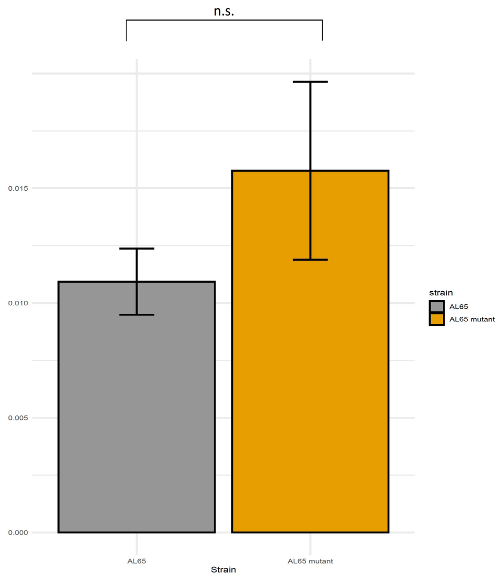

Fig S1. (A) Biofilm formation of AL65 and *tssM* mutant on abiotic surface quantified by measuring the absorbance (590 nm) of crystal violet. The strains were grown for 24 and 48 hours in XVM2 medium. The biofilm formed on the abiotic surface was stained with 0.1% crystal violet, excess stain was washed with ddH_2O and air dried. The crystal violet stain was solubilized with 40% methanol and the absorbance was measured at 570 nm. The error bar indicates standard deviation. Eight biological replicates of each strain were performed. (B) EPS production by the wild type and mutant strain. The error bar indicates standard deviation. Three biological replicates of each strain were performed. n.s. denotes not significant.

Table S1 (A) Complete list of downregulated genes in the mutant strain post 8 hours of growth in XVM2 medium

**locus tag**	**Annotation**	**log2FC**	**pvalue**	**padj**
E2P69_RS09405	type VI secretion system membrane subunit TssM	-6.701075531	1.82E-19	2.00E-18
E2P69_RS18255	methyl-accepting chemotaxis protein	-4.851110329	0	0
E2P69_RS18260	Chemotaxis protein CheW	-4.751087684	1.33E-168	8.50E-167
E2P69_RS20830	transporter	-4.619554781	7.65E-180	5.11E-178
E2P69_RS18265	EAL domain-containing response regulator	-4.616059685	0	0
E2P69_RS18245	STAS domain containing protein- also annotated as anti-anti-sigma factor	-4.474827149	0	0
E2P69_RS20895	chemotaxis protein CheW	-4.470638722	0	0
E2P69_RS02630	RebB family R body protein	-4.444374539	0	0
E2P69_RS18250	chemotaxis protein CheA	-4.37310124	0	0
E2P69_RS18270	SAM-dependent methyltransferase	-4.341460604	9.24E-253	7.97E-251
E2P69_RS20815	hypothetical protein	-4.3330243	1.11E-202	7.89E-201
E2P69_RS20845	MCP four helix bundle domain-containing protein	-4.320927557	0	0
E2P69_RS20910	chemotaxis protein CheR	-4.238746487	0	0
E2P69_RS20900	hypothetical protein	-4.216544817	0	0
E2P69_RS09150	purine-binding chemotaxis protein CheW	-4.15826063	0	0
E2P69_RS09155	MCP four helix bundle domain-containing protein	-4.081883544	0	0
E2P69_RS17280	EAL domain-containing protein	-4.051732322	0	0
E2P69_RS20905	MCP four helix bundle domain-containing protein	-4.025240913	0	0
E2P69_RS17295	chemotaxis protein	-4.005680124	0	0
E2P69_RS22515	response regulator	-4.00310479	0	0
E2P69_RS17180	flagellin flgL	-3.998847896	0	0
E2P69_RS17255	flagellar biosynthesis anti-sigma factor FlgM	-3.961931285	0	0
E2P69_RS22510	chemotaxis protein CheA	-3.950235556	0	0
E2P69_RS10740	hypothetical protein	-3.907031921	0	0
E2P69_RS22520	STAS domain-containing protein	-3.865265257	0	0
E2P69_RS15920	hypothetical protein	-3.843416711	0	0
E2P69_RS17260	flagella protein	-3.788126828	0	0
E2P69_RS17170	flagellar export chaperone FliS	-3.673064836	0	0
E2P69_RS22535	flagellar motor protein MotD	-3.602913258	0	0
E2P69_RS12285	cache domain-containing protein	-3.532192877	0	0
E2P69_RS06515	diguanylate cyclase	-3.515521738	0	0
E2P69_RS17290	hypothetical protein	-3.483189733	4.72E-71	1.76E-69
E2P69_RS18275	chemotaxis response regulator protein-glutamate methylesterase	-3.439889026	4.09E-279	3.85E-277

E2P69_RS22540	flagellar motor protein	-3.407540231	0	0
E2P69_RS03320	methyl-accepting chemotaxis protein	-3.364312844	0	0
E2P69_RS20920	chemotaxis response regulator protein-glutamate methylesterase	-3.329126753	3.10E-214	2.38E-212
E2P69_RS22530	ParA family protein	-3.216383494	1.08E-239	8.97E-238
E2P69_RS22525	chemotaxis protein CheW	-3.175511143	5.32E-217	4.16E-215
E2P69_RS20840	MCP four helix bundle domain-containing protein	-3.148165515	0	0
E2P69_RS04575	flagellar motor stator protein MotA	-3.143161066	0	0
E2P69_RS04570	flagellar motor protein MotB	-3.068792412	8.28E-245	7.00E-243
E2P69_RS12300	GGDEF domain-containing protein	-3.068555018	1.66E-106	8.00E-105
E2P69_RS16355	GGDEF domain-containing protein	-3.03243158	0	0
E2P69_RS10895	molecular chaperone	-3.031721361	1.55E-147	8.69E-146
E2P69_RS13670	EAL domain-containing protein	-2.989131962	3.72E-183	2.52E-181
E2P69_RS18845	hypothetical protein	-2.984598856	7.45E-123	3.76E-121
E2P69_RS20865	HAMP domain-containing protein	-2.981830408	7.44E-176	4.89E-174
E2P69_RS20820	cache domain-containing protein	-2.934425886	2.88E-206	2.13E-204
E2P69_RS20880	Cache 3/Cache 2 fusion domain-containing protein	-2.893352659	1.72E-302	1.74E-300
E2P69_RS20890	flagellar brake protein	-2.844612861	1.66E-136	8.83E-135
E2P69_RS17245	chemotaxis protein CheV	-2.781507291	0	0
E2P69_RS17160	PilZ domain-containing protein	-2.779298933	2.63E-280	2.53E-278
E2P69_RS17165	hypothetical protein	-2.75737704	8.93E-204	6.49E-202
E2P69_RS10890	hypothetical protein	-2.749676798	7.20E-68	2.59E-66
E2P69_RS22065	chemotaxis response regulator CheY	-2.658988103	0	0
E2P69_RS06725	PAS domain-containing methyl-accepting chemotaxis protein	-2.644878897	2.80E-259	2.52E-257
E2P69_RS20915	chemoreceptor glutamine deamidase CheD	-2.63406735	5.29E-162	3.13E-160
E2P69_RS19000	EAL domain-containing protein	-2.633325526	5.14E-161	3.00E-159
E2P69_RS22070	protein phosphatase CheZ	-2.615559284	1.02E-257	8.98E-256
E2P69_RS22060	RNA polymerase sigma factor FliA	-2.613170061	7.18E-225	5.72E-223
E2P69_RS17175	flagellar filament capping protein FliD	-2.564350175	0	0
E2P69_RS03015	ricin-type beta-trefoil lectin domain protein	-2.497385998	0	0
E2P69_RS19525	hypothetical protein	-2.485436938	3.31E-278	3.05E-276
E2P69_RS15820	roadblock/LC7 domain-containing protein	-2.412428544	6.73E-86	2.87E-84
E2P69_RS22055	MinD/ParA family protein	-2.390969006	3.45E-208	2.60E-206

E2P69_RS22075	Hpt domain-containing protein	-2.355473151	7.54E-169	4.88E-167
E2P69_RS21675	HD-GYP domain-containing protein	-2.275340113	1.06E-164	6.49E-163
E2P69_RS20850	CHASE3 domain-containing protein	-2.246640146	1.15E-126	5.88E-125
E2P69_RS22695	chemotaxis protein CheW	-2.224056024	1.54E-105	7.33E-104
E2P69_RS15825	hypothetical protein	-2.221689663	2.62E-192	1.84E-190
E2P69_RS20825	methyl-accepting chemotaxis protein	-2.188357292	3.67E-162	2.20E-160
E2P69_RS22035	bifunctional diguanylate cyclase/phosphodiesterase	-2.150998518	1.56E-86	6.80E-85
E2P69_RS02625	hypothetical protein	-2.131480013	6.25E-153	3.59E-151
E2P69_RS20875	MCP four helix bundle domain-containing protein	-2.056181233	4.63E-73	1.78E-71
E2P69_RS19765	pilin PilA	-1.911938451	1.79E-300	1.77E-298
E2P69_RS17185	flagellar hook-associated protein FlgL	-1.906683532	6.10E-238	4.95E-236
E2P69_RS20870	MCP four helix bundle domain-containing protein	-1.882889432	1.08E-56	3.17E-55
E2P69_RS12180	GGDEF domain-containing protein	-1.84994444	1.52E-30	2.51E-29
E2P69_RS22030	GGDEF domain-containing protein	-1.810513339	6.71E-144	3.71E-142
E2P69_RS11075	hypothetical protein	-1.709678822	1.59E-21	1.86E-20
E2P69_RS10550	GGDEF domain-containing protein	-1.693429255	9.45E-95	4.26E-93
E2P69_RS09910	methyl-accepting chemotaxis protein	-1.677941376	5.45E-99	2.54E-97
E2P69_RS17630	S8 family serine peptidase	-1.654436361	1.71E-49	4.39E-48
E2P69_RS11025	alginate export family protein	-1.631504888	5.21E-102	2.45E-100
E2P69_RS17190	flagellar hook-associated protein FlgK	-1.614157678	3.52E-168	2.21E-166
E2P69_RS00220	glycoside hydrolase family 5 protein	-1.55835815	1.30E-85	5.49E-84
E2P69_RS12280	PAS domain S-box protein	-1.497485017	4.88E-110	2.38E-108
E2P69_RS06485	OFA family MFS transporter	-1.491897391	1.21E-165	7.46E-164
E2P69_RS17195	flagellar assembly peptidoglycan hydrolase FlgJ	-1.450375232	8.75E-89	3.90E-87
E2P69_RS14220	group 1 truncated hemoglobin	-1.442008143	1.43E-28	2.21E-27
E2P69_RS06195	DUF5597 domain-containing protein	-1.425250657	3.03E-58	9.02E-57
E2P69_RS08385	Cache 3/Cache 2 fusion domain-containing protein	-1.415565201	1.52E-141	8.31E-140
E2P69_RS17225	flagellar hook protein FlgE	-1.39852265	4.40E-59	1.37E-57
E2P69_RS14205	methylamine utilization protein	-1.347870073	3.98E-54	1.13E-52
E2P69_RS14215	DUF3034 family protein	-1.331909762	5.28E-47	1.34E-45
E2P69_RS14485	TcpQ domain-containing protein	-1.324331209	1.53E-67	5.45E-66
E2P69_RS19570	S8 family serine peptidase	-1.312085896	3.03E-18	3.14E-17
E2P69_RS17220	flagellar basal-body rod protein FlgF	-1.26676339	5.27E-41	1.17E-39
E2P69_RS08390	hypothetical protein	-1.264462958	5.48E-52	1.48E-50

E2P69_RS04870	hypothetical protein	-1.263580941	1.16E-61	3.68E-60
E2P69_RS06495	FdhF/YdeP family oxidoreductase	-1.261224178	1.85E-122	9.21E-121
E2P69_RS17200	flagellar basal body P-ring protein FlgI	-1.243582151	3.48E-70	1.29E-68
E2P69_RS06200	hypothetical protein	-1.209503906	1.52E-22	1.87E-21
E2P69_RS15500	non-ribosomal peptide synthetase	-1.206996623	1.95E-137	1.05E-135
E2P69_RS05330	hypothetical protein	-1.198239076	1.08E-32	1.94E-31
E2P69_RS16890	hypothetical protein	-1.188295271	2.68E-69	9.84E-68
E2P69_RS14210	EAL domain-containing protein	-1.186257814	7.30E-69	2.65E-67
E2P69_RS06510	bacteriohemerythrin	-1.185809271	3.92E-50	1.03E-48
E2P69_RS17230	flagellar basal body rod modification protein FlgD	-1.133768163	1.11E-58	3.37E-57
E2P69_RS05890	glycoside hydrolase family 97 protein	-1.129546123	4.92E-22	5.96E-21
E2P69_RS01440	organic hydroperoxide resistance protein	-1.121676698	1.44E-62	4.73E-61
E2P69_RS22050	flagellar biosynthesis protein FlhF	-1.115426616	1.16E-42	2.74E-41
E2P69_RS02000	helix-hairpin-helix domain-containing protein	-1.114595105	7.94E-79	3.29E-77
E2P69_RS02895	HAMP domain-containing protein	-1.096522664	6.33E-32	1.10E-30
E2P69_RS18550	hypothetical protein	-1.090595519	8.35E-67	2.96E-65
E2P69_RS06490	formate dehydrogenase accessory sulfurtransferase FdhD	-1.08443221	1.04E-58	3.19E-57
E2P69_RS22045	flagellar biosynthesis protein FlhA	-1.082815951	1.15E-50	3.07E-49
E2P69_RS02705	response regulator	-1.061790282	2.33E-42	5.40E-41
E2P69_RS19010	cellulase family glycosylhydrolase	-1.05128453	5.48E-23	6.84E-22
E2P69_RS02690	Hpt domain-containing protein	-1.049957613	2.54E-115	1.25E-113
E2P69_RS02480	alpha/beta hydrolase	-1.048762411	4.96E-44	1.22E-42
E2P69_RS04875	hypothetical protein	-1.045780071	7.03E-22	8.41E-21
E2P69_RS17205	flagellar basal body L-ring protein FlgH	-1.04395146	3.16E-06	1.32E-05
E2P69_RS02700	chemotaxis protein CheW	-1.037919297	9.28E-43	2.21E-41
E2P69_RS17575	ferritin-like domain-containing protein	-1.032545943	4.11E-28	6.30E-27
E2P69_RS11305	hypothetical protein	-1.028578121	6.29E-10	3.79E-09
E2P69_RS10250	pilus assembly protein PilP	-1.025301444	3.96E-60	1.24E-58
E2P69_RS17035	flagellar motor switch protein FliN	-1.008999299	3.28E-26	4.66E-25
E2P69_RS15410	porin	-1.006693735	7.50E-65	2.55E-63
E2P69_RS17235	flagellar basal body rod protein FlgC	-1.00254446	3.87E-35	7.25E-34
E2P69_RS15495	glycosyltransferase family 1 protein	-1.000653529	1.17E-51	3.14E-50
E2P69_RS17250	flagellar basal body P-ring formation protein FlgA	-0.993969029	2.22E-30	3.61E-29

E2P69_RS17565	stress-induced protein	-0.970383888	3.06E-32	5.38E-31
E2P69_RS14500	TrbI/VirB10 family protein	-0.964429201	5.96E-54	1.67E-52
E2P69_RS17845	EAL domain-containing protein	-0.954467495	6.92E-12	4.78E-11
E2P69_RS02685	hypothetical protein	-0.952725613	6.94E-31	1.17E-29
E2P69_RS19420	P-type conjugative transfer protein TrbJ	-0.948538145	4.43E-71	1.67E-69
E2P69_RS14505	P-type DNA transfer ATPase VirB11	-0.948285693	1.29E-65	4.48E-64
E2P69_RS17055	flagellar export protein FliJ	-0.939031361	1.80E-25	2.49E-24
E2P69_RS09010	YaeQ family protein	-0.934548279	6.86E-13	5.09E-12
E2P69_RS14725	hypothetical protein	-0.923057226	1.73E-37	3.46E-36
E2P69_RS17155	response regulator transcription factor	-0.922342032	2.44E-25	3.37E-24
E2P69_RS18915	hypothetical protein	-0.920236041	2.56E-64	8.61E-63
E2P69_RS15260	amidohydrolase	-0.902457883	3.96E-25	5.38E-24
E2P69_RS10240	PilN domain-containing protein	-0.901912295	4.90E-42	1.11E-40
E2P69_RS07935	FKBP-type peptidyl-prolyl cis-trans isomerase	-0.896012364	5.96E-06	2.40E-05
E2P69_RS01325	putative modified peptide	-0.893792905	2.24E-19	2.45E-18
E2P69_RS06215	TonB-dependent receptor	-0.884303359	8.83E-13	6.47E-12
E2P69_RS00865	phenylalanine 4-monooxygenase	-0.881445931	2.58E-16	2.36E-15
E2P69_RS13225	DUF3228 family protein	-0.875661918	6.91E-11	4.43E-10
E2P69_RS20230	sce7725 family protein	-0.874860706	2.33E-62	7.59E-61
E2P69_RS19155	transcriptional regulator	-0.8737213	1.24E-46	3.13E-45
E2P69_RS14770	hypothetical protein	-0.870833234	4.47E-15	3.81E-14
E2P69_RS00675	hypothetical protein	-0.870400474	1.39E-42	3.25E-41
E2P69_RS22650	hypothetical protein	-0.867471645	2.47E-28	3.81E-27
E2P69_RS17030	flagellar biosynthetic protein FliO	-0.86660192	7.40E-28	1.12E-26
E2P69_RS17065	flagellar assembly protein FliH	-0.864809961	3.43E-25	4.69E-24
E2P69_RS02695	methyl-accepting chemotaxis protein	-0.863577843	7.01E-40	1.51E-38
E2P69_RS17585	DUF1349 domain-containing protein	-0.862140542	1.13E-18	1.18E-17
E2P69_RS18455	metalloregulator ArsR/SmtB family transcription factor	-0.858602952	4.39E-26	6.20E-25
E2P69_RS12125	SPFH domain-containing protein	-0.854698912	7.31E-08	3.72E-07
E2P69_RS10580	hypothetical protein	-0.851508628	2.90E-07	1.38E-06
E2P69_RS11405	response regulator	-0.847915111	5.17E-38	1.05E-36
E2P69_RS02940	peptidoglycan-associated lipoprotein Pal	-0.847281498	1.55E-39	3.30E-38
E2P69_RS19315	hypothetical protein	-0.846770539	2.09E-29	3.29E-28
E2P69_RS20120	CD225/dispanin family protein	-0.842557244	1.80E-17	1.76E-16
E2P69_RS10255	type IV pilus secretin PilQ family protein	-0.837361723	1.57E-63	5.26E-62
E2P69_RS02680	chemotaxis protein CheW	-0.835230279	1.98E-14	1.63E-13
E2P69_RS10245	type 4a pilus biogenesis protein PilO	-0.834732649	5.70E-43	1.36E-41
E2P69_RS01330	putative peptide maturation	-0.833420196	8.42E-15	7.07E-14

	dehydrogenase			
E2P69_RS08140	folate-binding protein YgfZ	-0.830178467	1.41E-19	1.56E-18
E2P69_RS09520	1-acyl-sn-glycerol-3-phosphate acyltransferase	-0.825259953	4.99E-20	5.56E-19
E2P69_RS10870	spore coat protein U domain-containing protein	-0.824050383	8.57E-50	2.22E-48
E2P69_RS07260	LLM class flavin-dependent oxidoreductase	-0.821657044	4.02E-22	4.90E-21
E2P69_RS11710	ATP-binding protein	-0.819752635	4.32E-13	3.26E-12
E2P69_RS17025	flagellar type III secretion system pore protein FliP	-0.810500832	2.96E-44	7.33E-43
E2P69_RS01360	hypothetical protein	-0.806353861	6.82E-15	5.74E-14
E2P69_RS10235	pilus assembly protein PilM	-0.804045034	1.42E-41	3.17E-40
E2P69_RS22020	diguanylate cyclase	-0.8039877	1.21E-27	1.80E-26
E2P69_RS18910	MASE1 domain-containing protein	-0.802284609	4.93E-50	1.28E-48
E2P69_RS17050	flagellar hook-length control protein FliK	-0.798880413	7.71E-29	1.20E-27
E2P69_RS11205	MFS transporter	-0.797160182	8.71E-28	1.31E-26
E2P69_RS22945	hypothetical protein	-0.796365532	1.21E-58	3.66E-57
E2P69_RS08725	cytochrome ubiquinol oxidase subunit I	-0.79195508	5.92E-31	1.00E-29
E2P69_RS08250	hypothetical protein	-0.791207532	5.96E-33	1.08E-31
E2P69_RS18460	acyl-CoA dehydrogenase family protein	-0.788921457	1.17E-20	1.30E-19
E2P69_RS07135	ribosome assembly RNA-binding protein YhbY	-0.788485748	3.67E-26	5.21E-25
E2P69_RS17060	FliI/YscN family ATPase	-0.784492859	9.59E-28	1.43E-26
E2P69_RS01550	hypothetical protein	-0.783690834	1.31E-42	3.07E-41
E2P69_RS11720	STAS domain-containing protein	-0.783566143	2.68E-38	5.52E-37
E2P69_RS12250	alpha,alpha-trehalase TreA	-0.782778136	5.12E-21	5.81E-20
E2P69_RS19955	endonuclease	-0.768414892	7.21E-25	9.57E-24
E2P69_RS19090	glyoxalase/bleomycin resistance/dioxygenase family protein	-0.76727419	9.92E-07	4.42E-06
E2P69_RS17070	flagellar motor switch protein FliG	-0.76534777	2.63E-37	5.18E-36
E2P69_RS17595	autotransporter-associated beta strand repeat-containing protein	-0.757665608	7.42E-54	2.06E-52
E2P69_RS15430	zinc-finger domain-containing protein	-0.752538551	1.85E-18	1.93E-17
E2P69_RS02710	twitching motility response regulator PilG	-0.750005988	3.68E-48	9.42E-47
E2P69_RS00225	glycoside hydrolase family 5 protein	-0.748690934	2.42E-27	3.57E-26
E2P69_RS09525	hypothetical protein	-0.747702615	1.70E-06	7.40E-06
E2P69_RS17120	SDR family oxidoreductase	-0.747365917	1.74E-24	2.30E-23
E2P69_RS11665	chemotaxis protein CheB	-0.742194548	0.000108329	0.000363408
E2P69_RS17115	SDR family oxidoreductase	-0.734230732	1.38E-17	1.37E-16
E2P69_RS17075	flagellar M-ring protein FliF	-0.732156202	3.44E-38	7.05E-37
E2P69_RS10570	efflux RND transporter	-0.730761638	7.94E-08	4.02E-07

	periplasmic adaptor subunit			
E2P69_RS11660	chemotaxis protein CheR	-0.728098632	1.98E-16	1.83E-15
E2P69_RS08115	hypothetical protein	-0.727495188	4.07E-10	2.50E-09
E2P69_RS08130	succinate dehydrogenase, cytochrome b556 subunit	-0.726091912	7.61E-24	9.85E-23
E2P69_RS10175	ribonuclease PH	-0.723634107	1.78E-09	1.03E-08
E2P69_RS12890	S-(hydroxymethyl)glutathione dehydrogenase/class III alcohol dehydrogenase	-0.721594953	1.60E-16	1.49E-15
E2P69_RS08110	succinate dehydrogenase iron-sulfur subunit	-0.719510534	1.61E-26	2.32E-25
E2P69_RS19685	nucleoside diphosphate kinase regulator	-0.714348796	5.70E-12	3.95E-11
E2P69_RS14490	type IV secretion system protein	-0.712110225	8.52E-31	1.43E-29
E2P69_RS11550	tryptophan 2,3-dioxygenase	-0.709758357	3.02E-15	2.60E-14
E2P69_RS11555	VOC family protein	-0.705999802	7.10E-19	7.54E-18
E2P69_RS15600	slipin family protein	-0.70482564	4.24E-10	2.60E-09
E2P69_RS20860	MCP four helix bundle domain-containing protein	-0.704705624	7.69E-07	3.49E-06
E2P69_RS12740	GspH/FimT family pseudopilin	-0.704549751	0.000504067	0.0014981
E2P69_RS20165	hypothetical protein	-0.70433844	2.51E-14	2.06E-13
E2P69_RS18350	DUF2974 domain-containing protein	-0.703047064	7.04E-24	9.15E-23
E2P69_RS08195	c-type cytochrome	-0.699542725	3.41E-19	3.68E-18
E2P69_RS08125	succinate dehydrogenase, hydrophobic membrane anchor protein	-0.699344482	5.52E-24	7.22E-23
E2P69_RS02075	BolA/IbaG family iron-sulfur metabolism protein	-0.699338527	2.15E-11	1.44E-10
E2P69_RS22645	hypothetical protein	-0.699287158	7.41E-18	7.46E-17
E2P69_RS20750	isoprenylcysteine carboxylmethyltransferase family protein	-0.698075881	1.86E-13	1.43E-12
E2P69_RS09975	adenylate kinase	-0.697910817	2.22E-31	3.79E-30
E2P69_RS17095	FkbM family methyltransferase	-0.696059366	4.27E-32	7.43E-31
E2P69_RS06995	hypothetical protein	-0.692742932	5.47E-14	4.41E-13
E2P69_RS01885	DUF3861 domain-containing protein	-0.691155506	2.88E-05	0.000105899
E2P69_RS01855	PilT/PilU family type 4a pilus ATPase	-0.689178948	7.04E-39	1.48E-37
E2P69_RS17105	Rieske 2Fe-2S domain-containing protein	-0.684522511	1.48E-23	1.88E-22
E2P69_RS10055	tetratricopeptide repeat protein	-0.683426952	1.17E-08	6.48E-08
E2P69_RS04965	phasin family protein	-0.683426121	4.81E-28	7.35E-27
E2P69_RS10575	SDR family oxidoreductase	-0.682489217	4.43E-05	0.000157675
E2P69_RS06500	hypothetical protein	-0.679418092	2.54E-07	1.21E-06
E2P69_RS02470	TonB-dependent receptor	-0.678397652	6.82E-28	1.04E-26
E2P69_RS14510	lytic transglycosylase domain-containing protein	-0.678199708	2.82E-29	4.43E-28
E2P69_RS22660	transposase	-0.676009022	6.10E-05	0.000213486

E2P69_RS14525	VirB4 family type IV secretion/conjugal transfer ATPase	-0.675355466	2.27E-37	4.52E-36
E2P69_RS05855	hypothetical protein	-0.674623578	9.17E-30	1.47E-28
E2P69_RS11715	STAS domain-containing protein	-0.674487602	3.27E-15	2.80E-14
E2P69_RS01765	hypothetical protein	-0.673648772	3.10E-15	2.67E-14
E2P69_RS01315	GGDEF domain-containing protein	-0.673438727	3.81E-21	4.36E-20
E2P69_RS10685	carbonic anhydrase	-0.671611049	8.92E-14	7.12E-13
E2P69_RS22185	hypothetical protein	-0.670276476	3.12E-19	3.38E-18
E2P69_RS01185	3-oxoacyl-ACP synthase III	-0.669518461	1.64E-22	2.01E-21
E2P69_RS01860	type IV pilus twitching motility protein PilT	-0.662048872	7.95E-32	1.37E-30
E2P69_RS17125	ketoacyl-ACP synthase III	-0.657937107	8.51E-32	1.46E-30
E2P69_RS00945	hypothetical protein	-0.65706523	1.38E-12	9.89E-12
E2P69_RS10910	GGDEF domain-containing protein	-0.653133334	4.33E-27	6.31E-26
E2P69_RS09245	rRNA maturation RNase YbeY	-0.650293363	2.60E-06	1.10E-05
E2P69_RS10225	hypothetical protein	-0.650051458	2.51E-19	2.73E-18
E2P69_RS13675	pseudouridine synthase	-0.641018135	7.42E-12	5.10E-11
E2P69_RS03075	ATP-binding cassette domain-containing protein	-0.639739265	4.21E-11	2.75E-10
E2P69_RS09205	ubiquinol-cytochrome c reductase iron-sulfur subunit	-0.63451142	2.17E-17	2.09E-16
E2P69_RS13920	DUF4870 domain-containing protein	-0.63434785	1.14E-23	1.46E-22
E2P69_RS05060	NAD-dependent epimerase/dehydratase family protein	-0.632336843	2.76E-16	2.52E-15
E2P69_RS13235	sulfotransferase domain-containing protein	-0.631516755	2.94E-25	4.04E-24
E2P69_RS21845	hypothetical protein	-0.627444702	0.011158816	0.025098249
E2P69_RS11535	SDR family oxidoreductase	-0.622776913	1.26E-07	6.20E-07
E2P69_RS19870	hypothetical protein	-0.622413384	5.80E-07	2.66E-06
E2P69_RS00325	DUF1868 domain-containing protein	-0.621663604	7.56E-28	1.14E-26
E2P69_RS19730	type IV-A pilus assembly ATPase PilB	-0.621341913	1.29E-21	1.52E-20
E2P69_RS18450	homocysteine S-methyltransferase family protein	-0.620798249	8.69E-19	9.16E-18
E2P69_RS06145	FAD-binding oxidoreductase	-0.619509051	0.011543186	0.025864478
E2P69_RS14105	GDP-mannose pyrophosphatase NudK	-0.619323178	0.000260142	0.000814638
E2P69_RS17100	UDP-3-O-(3-hydroxymyristoyl)glucosamine N-acyltransferase	-0.618490841	1.28E-15	1.13E-14
E2P69_RS13145	DUF721 domain-containing protein	-0.618386439	2.15E-17	2.08E-16
E2P69_RS05065	SDR family oxidoreductase	-0.617833243	2.02E-11	1.35E-10
E2P69_RS10315	TonB-dependent receptor	-0.617501219	2.93E-26	4.20E-25
E2P69_RS15005	TonB-dependent receptor	-0.61562502	4.25E-23	5.32E-22

E2P69_RS08320	tRNA adenosine(34) deaminase TadA	-0.615136251	0.000385024	0.001167756
E2P69_RS11545	FAD-dependent monooxygenase	-0.614677542	1.18E-10	7.43E-10
E2P69_RS01990	EF-hand domain-containing protein	-0.613403921	7.43E-08	3.77E-07
E2P69_RS07445	circadian clock protein KaiC	-0.612138762	1.20E-22	1.48E-21
E2P69_RS10560	SDR family oxidoreductase	-0.611766869	1.47E-05	5.63E-05
E2P69_RS00330	TonB-dependent receptor	-0.611306809	5.51E-25	7.42E-24
E2P69_RS08120	succinate dehydrogenase flavoprotein subunit	-0.611004846	2.85E-27	4.18E-26
E2P69_RS11080	hypothetical protein	-0.610773283	9.82E-05	0.000330546
E2P69_RS15935	catalase KatB	-0.608618819	4.90E-07	2.27E-06
E2P69_RS04995	PilT/PilU family type 4a pilus ATPase	-0.608599157	5.68E-22	6.85E-21
E2P69_RS16915	glycine C-acetyltransferase	-0.607494815	1.14E-10	7.17E-10
E2P69_RS19160	single-stranded DNA-binding protein	-0.607291539	1.37E-14	1.13E-13
E2P69_RS01770	hypothetical protein	-0.605945798	2.33E-07	1.12E-06
E2P69_RS15255	amidohydrolase family protein	-0.60593573	8.10E-13	5.96E-12
E2P69_RS17875	glycoside hydrolase family 92 protein	-0.605243574	4.37E-17	4.18E-16
E2P69_RS09865	3-isopropylmalate dehydratase large subunit	-0.604113309	8.84E-33	1.60E-31
E2P69_RS00565	hypothetical protein	-0.601748566	2.53E-06	1.07E-05
E2P69_RS12150	hypothetical protein	-0.599258143	0.006511665	0.015337025
E2P69_RS16420	alpha-2-macroglobulin family protein	-0.598641823	8.86E-28	1.33E-26
E2P69_RS11655	response regulator	-0.597863539	4.73E-18	4.84E-17
E2P69_RS02500	serine hydrolase	-0.59643969	2.30E-24	3.03E-23
E2P69_RS17110	NeuD/PglB/VioB family sugar acetyltransferase	-0.595672493	6.93E-18	6.98E-17
E2P69_RS16195	4-hydroxyphenylpyruvate dioxygenase	-0.591844615	1.93E-21	2.24E-20
E2P69_RS18545	OprO/OprP family phosphate-selective porin	-0.591231264	6.19E-35	1.16E-33
E2P69_RS06395	hypothetical protein	-0.590545932	9.18E-18	9.19E-17
E2P69_RS02415	O-succinylhomoserine (thiol)-lyase	-0.590164667	5.05E-17	4.80E-16
E2P69_RS11540	aminotransferase class V-fold PLP-dependent enzyme	-0.589776805	5.21E-06	2.11E-05
E2P69_RS19425	TrbI/VirB10 family protein	-0.588144806	5.21E-25	7.05E-24
E2P69_RS17080	flagellar hook-basal body complex protein FliE	-0.587356272	1.95E-16	1.79E-15
E2P69_RS04910	PLP-dependent cysteine synthase family protein	-0.586421951	1.59E-12	1.13E-11
E2P69_RS16445	hypothetical protein	-0.584804586	1.05E-06	4.67E-06
E2P69_RS13170	methylenetetrahydrofolate reductase [NAD(P)H]	-0.584694102	1.80E-17	1.76E-16
E2P69_RS10555	efflux transporter outer membrane subunit	-0.584177869	1.60E-07	7.85E-07
E2P69_RS20160	hypothetical protein	-0.584098133	7.25E-10	4.35E-09
E2P69_RS11400	23S rRNA (uracil(1939)-C(5))-	-0.581440011	7.06E-09	3.94E-08

	methyltransferase RlmD			
E2P69_RS09550	hypothetical protein	-0.580884105	1.86E-07	9.03E-07
E2P69_RS09800	DNA/RNA non-specific endonuclease	-0.580387431	2.57E-11	1.71E-10

Table S1 (B) Complete list of upregulated genes in the mutant strain post 8 hours of growth in XVM2 medium

Locus tag	Annotation	log2FC	pvalue	padj
E2P69_RS14430	type IV pilus modification protein PilV	2.96297418	0	0
E2P69_RS14425	GspH/FimT family pseudopilin	2.950872967	0	0
E2P69_RS14435	PilW family protein	2.80799529	0	0
E2P69_RS14440	Tfp pilus assembly protein PilX	2.771259494	0	0
E2P69_RS14445	pilus assembly protein	2.55575927	0	0
E2P69_RS21395	hypothetical protein	2.285685401	7.46E-96	3.39E-94
E2P69_RS14460	GspH/FimT family pseudopilin	2.234376707	1.48E-25	2.06E-24
E2P69_RS02190	proline iminopeptidase-family hydrolase	2.11349252	8.28E-37	1.60E-35
E2P69_RS07055	AlpA family phage regulatory protein	1.896737565	5.52E-42	1.25E-40
E2P69_RS11815	GTPase ObgE	1.689703641	4.49E-74	1.75E-72
E2P69_RS01340	acetyl/propionyl/methylcrotonyl-CoA carboxylase subunit alpha	1.678524337	1.82E-86	7.86E-85
E2P69_RS10215	type B 50S ribosomal protein L31	1.595951785	5.67E-127	2.94E-125
E2P69_RS04415	hypothetical protein	1.577699121	1.75E-21	2.05E-20
E2P69_RS07050	hypothetical protein	1.532081417	1.53E-12	1.09E-11
E2P69_RS12845	protein=thiol:disulfide interchange protein DsbA/DsbL	1.530788474	3.71E-73	1.44E-71
E2P69_RS11355	FAD-dependent monooxygenase	1.523652726	6.67E-30	1.08E-28
E2P69_RS19905	TonB-dependent receptor	1.512692145	1.95E-183	1.35E-181
E2P69_RS11485	acyl-CoA dehydrogenase family protein	1.500446954	5.82E-53	1.60E-51
E2P69_RS00455	hypothetical protein	1.492940584	5.38E-06	2.17E-05
E2P69_RS01345	methylcrotonoyl-CoA carboxylase	1.488141378	2.22E-76	8.92E-75
E2P69_RS14450	type IV pilin protein PilE	1.45160388	1.37E-135	7.19E-134
E2P69_RS05290	TonB-dependent receptor	1.425024759	1.31E-53	3.62E-52
E2P69_RS01310	isocitrate lyase	1.412973854	1.27E-32	2.27E-31
E2P69_RS15655	hypothetical protein	1.371788659	0.013943741	0.030517126
E2P69_RS01405	MerC family mercury resistance protein	1.301031815	7.80E-16	6.98E-15
E2P69_RS09410	type VI secretion system-associated protein TagF cluster I	1.261705392	0.000539042	0.001587243
E2P69_RS06800	hypothetical protein	1.261156632	0.00043821	0.001312727
E2P69_RS01350	isovaleryl-CoA dehydrogenase	1.254401598	6.73E-59	2.08E-57
E2P69_RS05285	tryptophan 7-halogenase	1.251668148	2.00E-19	2.18E-18
E2P69_RS11745	DUF805 domain-containing protein	1.245247853	5.90E-77	2.39E-75
E2P69_RS12595	sulfite exporter TauE/SafE family	1.221441132	7.96E-12	5.45E-11
E2P69_RS19910	glycoside hydrolase family 2	1.212246544	2.36E-148	1.34E-146
E2P69_RS11480	enoyl-CoA hydratase	1.2030048	8.82E-12	6.03E-11
E2P69_RS22405	50S ribosomal protein L29 rpmC	1.198504208	1.70E-32	3.02E-31
E2P69_RS04970	6-carboxytetrahydropterin synthase QueD	1.177259428	3.90E-27	5.71E-26
E2P69_RS01400	SHOCT domain-containing protein	1.170120872	7.81E-32	1.35E-30
E2P69_RS22380	50S ribosomal protein L2 rplB	1.164976754	9.83E-82	4.11E-80
E2P69_RS10950	hypothetical protein	1.163354025	1.23E-30	2.05E-29
E2P69_RS15800	CsbD family protein	1.158509622	2.24E-50	5.94E-49

E2P69_RS22345	elongation factor Tu	1.15007717	9.33E-36	1.77E-34
E2P69_RS05420	FGGY-family carbohydrate kinase	1.148633955	2.51E-07	1.20E-06
E2P69_RS21010	single-stranded-DNA-specific exonuclease RecJ	1.148401937	5.60E-34	1.03E-32
E2P69_RS03620	hypothetical protein	1.129177667	0.015581948	0.033745954
E2P69_RS22105	DNA-directed RNA polymerase subunit beta rpoB	1.125258709	9.42E-67	3.31E-65
E2P69_RS04065	hypothetical protein	1.113549785	0.011861271	0.026505526
E2P69_RS03460	30S ribosomal protein S21 rpsU	1.109691053	5.45E-46	1.36E-44
E2P69_RS20590	hypothetical protein	1.106907796	2.40E-07	1.15E-06
E2P69_RS20585	8-amino-7-oxononanoate synthase	1.100862757	3.18E-71	1.21E-69
E2P69_RS22430	30S ribosomal protein S14 rpsN	1.092495981	2.26E-36	4.33E-35
E2P69_RS01395	ExeM/NucH family extracellular endonuclease	1.092129562	2.23E-65	7.63E-64
E2P69_RS22490	50S ribosomal protein L17 rplQ	1.09183861	2.43E-56	7.08E-55
E2P69_RS11805	murein biosynthesis integral membrane protein MurJ	1.091794605	3.60E-35	6.78E-34
E2P69_RS00250	hypothetical protein	1.089837352	1.16E-52	3.17E-51
E2P69_RS10965	acyl-ACP--UDP-N-acetylglucosamine O-acyltransferase	1.086840459	3.47E-42	7.94E-41
E2P69_RS11810	30S ribosomal protein S20 rpsT	1.085011643	2.09E-60	6.60E-59
E2P69_RS22365	50S ribosomal protein L3 rplC	1.079482982	4.38E-54	1.23E-52
E2P69_RS22465	preprotein translocase subunit SecY	1.07363408	5.25E-97	2.42E-95
E2P69_RS11350	ligase-associated DNA damage response endonuclease PdeM	1.062160398	5.42E-14	4.39E-13
E2P69_RS15710	RNA polymerase sigma factor RpoE	1.056859871	6.95E-13	5.14E-12
E2P69_RS01380	DUF3649 domain-containing protein	1.053361407	0.020311071	0.042542349
E2P69_RS21805	50S ribosomal protein L33 rpmG	1.051928997	2.85E-23	3.58E-22
E2P69_RS17980	SDR family oxidoreductase	1.049774493	1.68E-11	1.14E-10
E2P69_RS22360	30S ribosomal protein S10 rpsJ	1.048846754	2.36E-87	1.04E-85
E2P69_RS03455	GatB/YqeY domain-containing	1.047232179	2.54E-58	7.62E-57
E2P69_RS22370	50S ribosomal protein L4 rplD	1.045021494	3.60E-40	7.85E-39
E2P69_RS21800	50S ribosomal protein L28 rpmB	1.032727902	4.15E-74	1.64E-72
E2P69_RS22130	transcription termination/antitermination protein NusG	1.021590119	2.89E-63	9.59E-62
E2P69_RS22385	30S ribosomal protein rpsS	1.019604412	2.28E-38	4.72E-37
E2P69_RS22085	elongation factor G	1.006183448	8.39E-62	2.69E-60
E2P69_RS22395	30S ribosomal protein rpsC	1.003833808	3.75E-38	7.65E-37
E2P69_RS12640	hypothetical protein	1.001967262	1.05E-23	1.34E-22
E2P69_RS06850	HK97 family phage prohead protease	1.000515866	0.002991366	0.007598545
E2P69_RS12520	rod shape-determining protein MreC	0.998021732	2.39E-21	2.77E-20
E2P69_RS22110	50S ribosomal protein L7/L12	0.997872952	3.42E-50	9.04E-49
E2P69_RS14340	30S ribosome-binding factor RbfA	0.991032367	5.00E-18	5.09E-17
E2P69_RS15415	dicarboxylate/amino acid:cation symporter	0.984367561	3.84E-32	6.71E-31
E2P69_RS10975	ribonuclease HII	0.978637321	2.77E-23	3.49E-22
E2P69_RS22410	30S ribosomal protein S17	0.97302127	9.88E-58	2.92E-56
E2P69_RS11600	tRNA (guanosine(37)-N1)-methyltransferase TrmD	0.971042644	3.65E-37	7.16E-36
E2P69_RS11610	30S ribosomal protein	0.97020484	2.92E-77	1.20E-75
E2P69_RS21535	DUF2490 domain-containing protein	0.964599411	7.62E-22	9.04E-21
E2P69_RS22115	50S ribosomal protein L10	0.957239768	1.44E-30	2.39E-29

E2P69_RS19840	autotransporter domain-containing esterase	0.954512266	8.46E-38	1.70E-36
E2P69_RS05775	L-fucose:H+ symporter permease	0.947971169	6.96E-25	9.27E-24
E2P69_RS11475	enoyl-CoA hydratase/isomerase family protein	0.943076137	1.92E-09	1.11E-08
E2P69_RS11260	DUF1328 domain-containing protein	0.943045298	1.50E-32	2.68E-31
E2P69_RS04270	DUF2894 domain-containing protein	0.939192952	0.001458658	0.00396017
E2P69_RS22375	50S ribosomal protein L23	0.934295584	2.64E-40	5.79E-39
E2P69_RS22415	50S ribosomal protein L14	0.934163047	1.20E-54	3.47E-53
E2P69_RS22575	S8 family serine peptidase	0.929297266	4.21E-14	3.43E-13
E2P69_RS22420	50S ribosomal protein L24	0.92412605	5.48E-44	1.34E-42
E2P69_RS04525	fasciclin domain-containing protein	0.92248903	2.13E-42	4.96E-41
E2P69_RS22390	50S ribosomal protein L22	0.9185309	7.25E-21	8.18E-20
E2P69_RS05650	hypothetical protein	0.917208387	3.93E-34	7.27E-33
E2P69_RS06320	hypothetical protein	0.915865551	3.06E-26	4.37E-25
E2P69_RS11825	50S ribosomal protein L21	0.915402628	1.12E-74	4.47E-73
E2P69_RS19465	50S ribosomal protein L25/general stress protein Ctc	0.912472447	6.09E-62	1.97E-60
E2P69_RS00070	50S ribosomal protein L34	0.909315906	0.004143907	0.010255798
E2P69_RS15705	anti-sigma factor	0.908157795	8.53E-08	4.29E-07
E2P69_RS22100	DNA-directed RNA polymerase subunit beta	0.907420057	5.45E-55	1.58E-53
E2P69_RS22125	50S ribosomal protein L11	0.905193659	2.10E-65	7.25E-64
E2P69_RS00440	CsbD family protein: bacterial general stress response protei	0.903513683	4.32E-36	8.20E-35
E2P69_RS00855	cupin domain-containing protein	0.902459879	0.000198928	0.000636909
E2P69_RS22445	50S ribosomal protein L18	0.902088381	8.80E-42	1.97E-40
E2P69_RS16085	GNAT family N-acetyltransferase	0.89678331	8.34E-39	1.75E-37
E2P69_RS22400	50S ribosomal protein L16	0.891738118	5.06E-40	1.10E-38
E2P69_RS06810	DUF3168 domain-containing protein	0.891577872	9.76E-05	0.000328877
E2P69_RS08540	50S ribosomal protein L36	0.89039516	1.08E-17	1.07E-16
E2P69_RS22460	50S ribosomal protein L15	0.889368225	1.24E-25	1.74E-24
E2P69_RS11820	50S ribosomal protein L27	0.887603136	3.31E-44	8.16E-43
E2P69_RS21015	hypothetical protein	0.887348684	4.04E-21	4.61E-20
E2P69_RS10955	UDP-3-O-(3-hydroxymyristoyl)glucosamine N-acyltransferase	0.880215642	2.53E-37	5.01E-36
E2P69_RS22450	30S ribosomal protein S5	0.858367885	5.40E-41	1.19E-39
E2P69_RS04280	DUF802 domain-containing protein	0.858087011	1.70E-08	9.27E-08
E2P69_RS18785	50S ribosomal protein L9	0.856034615	2.08E-30	3.42E-29
E2P69_RS22135	preprotein translocase subunit SecE	0.855002899	9.24E-39	1.92E-37
E2P69_RS07035	recombination-associated protein RdgC	0.853927902	1.65E-05	6.28E-05
E2P69_RS12835	endonuclease/exonuclease/phosphatase family protein	0.851794738	1.59E-29	2.53E-28
E2P69_RS14345	tRNA pseudouridine(55) synthase TruB	0.850460086	4.96E-13	3.73E-12
E2P69_RS10970	lipid-A-disaccharide synthase	0.848371584	7.04E-23	8.76E-22
E2P69_RS13345	TonB-dependent hemoglobin/transferrin/lactoferrin family receptor	0.846791384	0.007160349	0.016755671
E2P69_RS04455	hypothetical protein	0.844432578	2.09E-08	1.12E-07
E2P69_RS04185	hypothetical protein	0.842483874	5.05E-05	0.000179143

E2P69_RS15635	DUF4142 domain-containing protein	0.841334477	1.29E-25	1.81E-24
E2P69_RS22120	50S ribosomal protein L1	0.830525983	1.62E-46	4.06E-45
E2P69_RS07905	DUF3300 domain-containing protein	0.823611244	8.28E-30	1.33E-28
E2P69_RS16500	aminodeoxychorismate synthase component I	0.820754808	5.61E-11	3.63E-10
E2P69_RS21390	hypothetical protein	0.816931725	2.10E-24	2.77E-23
E2P69_RS16130	M4 family metallopeptidase	0.815846735	3.80E-43	9.16E-42
E2P69_RS11335	hypothetical protein	0.813954826	0.00045757	0.001367757
E2P69_RS21030	hypothetical protein	0.81369805	8.56E-08	4.30E-07
E2P69_RS21330	carbon storage regulator CsrA	0.812420339	1.93E-23	2.45E-22
E2P69_RS07040	hypothetical protein	0.809388805	3.81E-06	1.57E-05
E2P69_RS21740	GlsB/YeaQ/YmgE family stress response membrane protein	0.801769179	4.97E-30	8.07E-29
E2P69_RS01810	glycine zipper 2TM domain-containing protein	0.798379658	9.32E-40	1.99E-38
E2P69_RS18840	membrane-bound PQQ-dependent dehydrogenase, glucose/quinate/shikimate family	0.796507089	1.86E-21	2.16E-20
E2P69_RS07590	type VI secretion system-associated protein TagF	0.794895763	0.016640526	0.035702588
E2P69_RS10410	TIGR00730 family Rossman fold protein	0.794614286	1.78E-19	1.96E-18
E2P69_RS04760	hypothetical protein	0.794610998	1.38E-06	6.05E-06
E2P69_RS05930	50S ribosomal protein L20	0.793208967	1.49E-54	4.27E-53
E2P69_RS01215	DUF1328 domain-containing protein	0.790153869	3.75E-10	2.31E-09
E2P69_RS04200	hypothetical protein	0.786784685	0.000116774	0.000388904
E2P69_RS01265	peptidyl-dipeptidase Dcp	0.783269323	2.33E-36	4.45E-35
E2P69_RS03920	hypothetical protein	0.78187841	0.003479358	0.008720495
E2P69_RS04285	DUF3348 domain-containing protein	0.771881954	5.60E-08	2.87E-07
E2P69_RS05780	L-fucose mutarotase	0.771607428	2.66E-08	1.41E-07
E2P69_RS15660	DUF3313 domain-containing protein	0.77050092	5.43E-11	3.52E-10
E2P69_RS03820	O-antigen ligase family protein	0.770184391	4.46E-11	2.90E-10
E2P69_RS06865	HNH endonuclease	0.766963532	0.004986652	0.012095842
E2P69_RS22455	50S ribosomal protein L30	0.764648619	5.26E-25	7.09E-24
E2P69_RS12525	rod shape-determining protein MreD	0.764550811	4.84E-07	2.25E-06
E2P69_RS04815	chorismate mutase	0.759586118	0.003882784	0.009649894
E2P69_RS10020	HAD-IA family hydrolase	0.758896438	0.003112466	0.007886818
E2P69_RS22440	50S ribosomal protein L6	0.757800925	2.59E-42	5.97E-41
E2P69_RS09320	hypothetical protein	0.75776001	7.76E-22	9.18E-21
E2P69_RS11835	acyl-CoA thioesterase	0.751372918	3.80E-16	3.45E-15
E2P69_RS22435	30S ribosomal protein S8	0.750967996	6.99E-29	1.09E-27
E2P69_RS07210	hypothetical protein	0.74902705	6.57E-26	9.23E-25
E2P69_RS19460	aminoacyl-tRNA hydrolase	0.7489387	1.04E-08	5.80E-08
E2P69_RS18995	sodium/sugar symporter	0.742960436	3.21E-15	2.75E-14
E2P69_RS11470	3-hydroxyisobutyrate dehydrogenase	0.737178254	3.81E-09	2.16E-08
E2P69_RS13990	FAD-dependent oxidoreductase	0.732904191	1.58E-13	1.23E-12
E2P69_RS05520	xylose isomerase XylA	0.731376094	1.10E-16	1.04E-15
E2P69_RS12265	hypothetical protein	0.730934584	5.68E-10	3.44E-09
E2P69_RS04450	response regulator transcription factor LytTR	0.727916567	5.81E-10	3.51E-09
E2P69_RS16280	efflux RND transporter periplasmic adaptor subunit	0.724336453	0.020566776	0.04303442
E2P69_RS18590	sulfurtransferase	0.722019141	5.37E-22	6.48E-21

E2P69_RS03990	phage tail tape measure protein	0.722007246	0.000608185	0.001766977
E2P69_RS19900	LacI family DNA-binding transcriptional regulator	0.717708798	2.00E-13	1.54E-12
E2P69_RS11595	50S ribosomal protein L19	0.714607591	2.16E-32	3.83E-31
E2P69_RS04210	NAD(P)H-dependent oxidoreductase	0.713288682	0.001746493	0.004674239
E2P69_RS12825	choline BCCT transporter BetT	0.709594812	2.41E-23	3.05E-22
E2P69_RS16925	rRNA pseudouridine synthase	0.708686908	1.19E-08	6.54E-08
E2P69_RS04730	outer membrane beta-barrel protein	0.705323668	4.97E-34	9.15E-33
E2P69_RS22425	50S ribosomal protein L5	0.703987535	4.06E-37	7.93E-36
E2P69_RS07980	hypothetical protein	0.703986109	8.04E-40	1.73E-38
E2P69_RS13630	DUF2007 domain-containing protein	0.700847968	1.44E-07	7.10E-07
E2P69_RS09065	N-acetylmuramoyl-L-alanine amidase	0.69969276	6.79E-30	1.09E-28
E2P69_RS12655	BON domain-containing protein	0.697901859	6.25E-44	1.52E-42
E2P69_RS22790	hypothetical protein	0.697504279	4.88E-18	4.98E-17
E2P69_RS16430	nucleoside hydrolase	0.6973253	2.11E-11	1.41E-10
E2P69_RS18510	RcnB family protein	0.694363833	3.06E-32	5.38E-31
E2P69_RS03605	LysM peptidoglycan-binding domain-containing protein	0.693319768	9.36E-10	5.53E-09
E2P69_RS12530	penicillin-binding protein 2	0.691023317	8.38E-15	7.04E-14
E2P69_RS11870	EF-hand domain-containing protein	0.690100328	1.48E-13	1.15E-12
E2P69_RS15720	cytochrome b	0.686576189	5.19E-10	3.15E-09
E2P69_RS18585	CoA pyrophosphatase	0.685935336	4.44E-19	4.77E-18
E2P69_RS05925	50S ribosomal protein L35	0.684910666	1.24E-26	1.80E-25
E2P69_RS06790	hypothetical protein	0.683316572	1.54E-08	8.42E-08
E2P69_RS13655	hypothetical protein	0.680913573	3.86E-05	0.00013912
E2P69_RS08735	cytochrome bd-I oxidase subunit CydX	0.679634159	2.45E-07	1.17E-06
E2P69_RS07790	sulfite exporter TauE/SafE family protein	0.676443833	5.81E-13	4.32E-12
E2P69_RS00245	NAD(P)-dependent alcohol dehydrogenase	0.675813183	2.97E-25	4.07E-24
E2P69_RS08770	hypothetical protein	0.675347028	1.20E-05	4.65E-05
E2P69_RS03815	glycosyltransferase family 39 protein	0.674592454	1.27E-11	8.65E-11
E2P69_RS11995	phosphatase PAP2 family protein	0.673962438	5.99E-39	1.27E-37
E2P69_RS01100	glycosyltransferase family 25 protein	0.672692223	5.29E-14	4.29E-13
E2P69_RS17610	efflux transporter outer membrane subunit	0.670748901	0.018380081	0.038910922
E2P69_RS07805	alpha/beta hydrolase	0.667146037	1.54E-10	9.61E-10
E2P69_RS05985	acyltransferase family protein-gum cluster	0.666110714	1.98E-29	3.13E-28
E2P69_RS05215	pectate lyase	0.663526031	0.004064397	0.01007105
E2P69_RS20700	PepSY domain-containing protein	0.663124659	4.06E-11	2.66E-10
E2P69_RS05720	PQQ-binding-like beta-propeller repeat protein	0.66184623	2.48E-17	2.39E-16
E2P69_RS18780	30S ribosomal protein S18	0.661508085	1.91E-15	1.66E-14
E2P69_RS11605	ribosome maturation factor RimM	0.661360766	9.18E-19	9.65E-18
E2P69_RS14465	excinuclease ABC subunit UvrB	0.660440874	9.50E-22	1.12E-20
E2P69_RS11210	alkene reductase	0.659460458	2.05E-06	8.84E-06
E2P69_RS15575	acyl-CoA desaturase	0.65917292	1.85E-36	3.56E-35
E2P69_RS07870	efflux RND transporter periplasmic adaptor subunit	0.656619082	0.00601069	0.01430344
E2P69_RS12045	carboxylesterase family protein	0.654108012	5.92E-11	3.81E-10
E2P69_RS15675	hypothetical protein	0.653494926	2.86E-14	2.34E-13

E2P69_RS20110	LysR family transcriptional regulator	0.652799511	0.001157501	0.003201286
E2P69_RS02950	cell envelope integrity protein TolA	0.65113024	5.74E-24	7.47E-23
E2P69_RS12980	hydrolase CocE/NonD family	0.650066476	1.85E-07	9.00E-07
E2P69_RS00815	TRAP transporter substrate-binding protein	0.649507149	1.00E-05	3.92E-05
E2P69_RS06845	phage major capsid protein	0.648573455	2.43E-06	1.04E-05
E2P69_RS00065	ribonuclease P protein component	0.646723094	2.65E-11	1.76E-10
E2P69_RS12635	glycine zipper 2TM domain-containing protein	0.646319778	2.29E-33	4.17E-32
E2P69_RS02285	MFS transporter	0.646133611	0.001919438	0.005094318
E2P69_RS07515	3-phosphoshikimate 1-carboxyvinyltransferase aroA	0.644968759	4.08E-19	4.40E-18
E2P69_RS06095	excinuclease Cho	0.644447476	0.002303181	0.006027844
E2P69_RS00875	TonB-dependent siderophore receptor	0.643813793	2.28E-06	9.78E-06
E2P69_RS05965	gumC family protein	0.643400129	5.19E-38	1.05E-36
E2P69_RS05935	phenylalanine--tRNA ligase subunit alpha PheS	0.643087535	6.75E-18	6.84E-17
E2P69_RS18090	TonB-dependent receptor	0.640979521	1.12E-08	6.21E-08
E2P69_RS06020	gum cluster cupin domain	0.640590211	3.08E-05	0.000112651
E2P69_RS16235	SH3 domain-containing protein	0.638832454	3.51E-05	0.000127264
E2P69_RS19925	3-deoxy-7-phosphoheptulonate synthase	0.636932916	1.40E-26	2.03E-25
E2P69_RS11360	cold-shock protein	0.635207614	5.62E-16	5.05E-15
E2P69_RS05980	acyltransferase family gum cluster	0.633368271	1.12E-34	2.08E-33
E2P69_RS06770	phage minor tail protein L	0.629158774	0.001127601	0.00312276
E2P69_RS20330	siderophore-interacting protein	0.628828818	3.09E-10	1.90E-09
E2P69_RS16690	peptidyl-prolyl cis-trans isomerase	0.627061023	4.61E-29	7.21E-28
E2P69_RS22820	hypothetical protein	0.626995346	1.93E-13	1.48E-12
E2P69_RS04550	DUF3297 family protein	0.625339509	2.67E-13	2.04E-12
E2P69_RS06755	DUF1983 domain-containing protein	0.625150745	2.79E-05	0.000102726
E2P69_RS06005	glycosyltransferase gum cluster	0.623053384	1.76E-27	2.60E-26
E2P69_RS09935	helix-turn-helix transcriptional regulator	0.622961371	7.37E-05	0.000254903
E2P69_RS14115	transglycosylase SLT domain-containing protein	0.621584212	4.91E-15	4.18E-14
E2P69_RS11800	bifunctional riboflavin kinase/FAD synthetase	0.616888321	1.49E-18	1.55E-17
E2P69_RS12035	hypothetical protein	0.616607912	6.68E-42	1.51E-40
E2P69_RS04445	histidine kinase	0.615571486	1.59E-15	1.39E-14
E2P69_RS03970	N-acetylmuramoyl-L-alanine amidase	0.615148405	8.37E-23	1.04E-21
E2P69_RS07045	hypothetical protein	0.613368182	0.0083777	0.019401235
E2P69_RS19915	glycoside hydrolase family 97 protein	0.612206889	1.63E-29	2.58E-28
E2P69_RS13610	DMT family transporter	0.61102592	1.54E-07	7.55E-07
E2P69_RS01335	S9 family peptidase	0.610487215	5.58E-12	3.88E-11
E2P69_RS03810	16S rRNA (cytosine(967)-C(5))-methyltransferase RsmB	0.610169266	4.69E-06	1.91E-05
E2P69_RS13550	AMP-binding protein	0.609051228	0.001865882	0.0049649
E2P69_RS08745	MFS transporter	0.603509097	0.002919705	0.0074454
E2P69_RS21510	hypothetical protein	0.603098143	6.15E-07	2.81E-06
E2P69_RS12315	glucans biosynthesis glucosyltransferase MdoH	0.6002566	2.26E-14	1.86E-13
E2P69_RS06835	phage portal protein	0.599239498	0.000316065	0.000973574
E2P69_RS03645	transcription termination factor Rho	0.59867652	3.75E-31	6.37E-30

E2P69_RS21325	hypothetical protein	0.596879102	1.73E-16	1.61E-15
E2P69_RS07775	YeeE/YedE family protein	0.595058607	4.52E-08	2.34E-07
E2P69_RS12840	thiol:disulfide interchange protein DsbA/DsbL	0.594196035	3.00E-22	3.67E-21
E2P69_RS08905	phytoene synthase	0.59199703	0.00030893	0.000953724
E2P69_RS17985	hypothetical protein	0.591429553	0.000796429	0.002267769
E2P69_RS12515	rod shape-determining protein	0.59134013	3.03E-21	3.49E-20
E2P69_RS12165	S9 family peptidase	0.589761899	7.74E-10	4.62E-09
E2P69_RS21450	type IV secretory pathway, TrbF protein	0.589599882	7.59E-13	5.60E-12
E2P69_RS22810	hypothetical protein	0.588929195	0.009914344	0.022543977
E2P69_RS14325	ribosome maturation factor RimP	0.588817221	4.34E-21	4.94E-20
E2P69_RS09230	tRNA (N6-isopentenyl adenosine(37)-C2)-methylthiotransferase MiaB	0.588589557	1.13E-16	1.07E-15
E2P69_RS04305	hypothetical protein	0.58824762	8.84E-05	0.000300788
E2P69_RS20335	histidine-type phosphatase	0.586233774	1.58E-12	1.12E-11
E2P69_RS03445	YihY/virulence factor BrkB family protein	0.585927745	2.87E-15	2.48E-14
E2P69_RS11930	DUF423 domain-containing protein	0.584935271	0.000210146	0.00067075
E2P69_RS07115	tRNA (adenosine(37)-N6)-dimethylallyltransferase MiaA	0.583791769	1.56E-09	9.16E-09
E2P69_RS07785	TIGR01244 family phosphatase	0.58284063	2.93E-10	1.81E-09
E2P69_RS14025	peptidase	0.582380875	3.44E-14	2.81E-13
E2P69_RS01505	FAD-dependent urate hydroxylase HpyO	0.582037423	0.001350537	0.003695691
E2P69_RS00460	M4 family metallopeptidase	0.581683938	0.000257773	0.000809055
E2P69_RS12080	hypothetical protein	0.581127622	4.66E-08	2.41E-07
E2P69_RS06685	DUF1275 domain-containing protein	0.58065151	0.000109751	0.000367582
E2P69_RS22210	SDR family NAD(P)-dependent oxidoreductase	0.580591285	1.25E-12	8.97E-12

Table S1 (C) Complete list of downregulated genes in the mutant strain post 16 hours of growth in XVM2 medium

Locus tag	Annotation	log2FC	pvalue	padj
E2P69_RS09405	type VI secretion system membrane subunit TssM	-8.749333873	2.52E-17	1.38E-16
E2P69_RS18245	STAS domain-containing protein	-2.748656239	3.36E-41	3.97E-40
E2P69_RS17255	flagellar biosynthesis anti-sigma factor FlgM	-2.478707727	3.18E-89	9.56E-88
E2P69_RS10740	hypothetical protein	-2.302659485	5.13E-246	8.17E-244
E2P69_RS17260	flagella protein FlgN	-2.161837749	1.48E-109	5.37E-108
E2P69_RS17240	flagellar basal body rod protein FlgB	-2.16139985	2.77E-73	6.33E-72
E2P69_RS22040	flagellar biosynthesis protein FlhB	-2.088719775	1.88E-162	1.42E-160
E2P69_RS22010	flagellar biosynthetic protein FliQ	-2.081108785	7.86E-159	5.61E-157
E2P69_RS17030	flagellar biosynthetic protein FliO	-2.043175216	2.65E-174	2.20E-172
E2P69_RS02660	VWA domain-containing protein	-1.961255536	1.70E-132	7.84E-131
E2P69_RS20895	chemotaxis protein CheW	-1.939924343	3.12E-39	3.46E-38
E2P69_RS17250	flagellar basal body P-ring formation protein FlgA	-1.936071221	4.43E-143	2.41E-141
E2P69_RS17210	flagellar basal-body rod protein FlgG	-1.904858951	2.11E-65	4.13E-64
E2P69_RS17080	flagellar hook-basal body complex protein FliE	-1.901537833	2.56E-152	1.68E-150
E2P69_RS22535	flagellar motor protein MotD	-1.894837148	3.13E-145	1.91E-143
E2P69_RS22520	STAS domain-containing protein	-1.867578603	4.95E-80	1.27E-78
E2P69_RS17295	chemotaxis protein	-1.842242524	7.91E-145	4.68E-143
E2P69_RS17235	flagellar basal body rod protein FlgC	-1.824353054	1.05E-55	1.70E-54
E2P69_RS22050	flagellar biosynthesis protein FlhF	-1.787733906	1.70E-94	5.32E-93
E2P69_RS10870	spore coat protein U domain-containing protein	-1.756526508	1.38E-194	1.46E-192
E2P69_RS17180	flagellin FlgL	-1.749155097	8.23E-91	2.56E-89
E2P69_RS20480	HrpB1 family type III secretion system apparatus protein	-1.744686452	0	0
E2P69_RS18255	methyl-accepting chemotaxis protein	-1.733148176	1.90E-39	2.12E-38
E2P69_RS20415	hypothetical protein	-1.728295791	0	0
E2P69_RS17045	flagellar basal body-associated FliL family protein	-1.718998264	3.57E-181	3.29E-179
E2P69_RS00220	glycoside hydrolase family 5 protein	-1.709734126	2.97E-114	1.14E-112
E2P69_RS20910	chemotaxis protein CheR	-1.703476569	1.26E-27	1.02E-26
E2P69_RS22540	flagellar motor protein	-1.696319679	7.53E-89	2.23E-87
E2P69_RS20530	serine kinase - type III?	-1.673770885	1.94E-279	3.83E-277
E2P69_RS18260	purine-binding chemotaxis protein CheW	-1.660458303	2.34E-10	8.77E-10
E2P69_RS18100	hypothetical protein	-1.64450811	3.57E-129	1.56E-127
E2P69_RS08365	hydrolase	-1.618546959	1.18E-51	1.76E-50
E2P69_RS20900	hypothetical protein	-1.618424078	2.29E-40	2.64E-39
E2P69_RS08370	helix-turn-helix transcriptional regulator	-1.578683165	4.23E-32	3.85E-31
E2P69_RS10890	hypothetical protein	-1.566026687	3.53E-19	2.10E-18
E2P69_RS01610	hypothetical protein	-1.556095567	4.69E-248	8.10E-246
E2P69_RS01615	LysR family transcriptional regulator	-1.54924591	4.99E-65	9.62E-64
E2P69_RS20475	type III secretion protein HrpB2	-1.53527165	5.55E-298	1.44E-295

E2P69_RS20500	type III secretion system cytoplasmic ring protein SctQ	-1.508658292	0	0
E2P69_RS18580	FKBP-type peptidyl-prolyl cis-trans isomerase	-1.503014453	0	0
E2P69_RS17175	flagellar filament capping protein FliD	-1.501824343	1.55E-166	1.21E-164
E2P69_RS17280	EAL domain-containing protein	-1.495540017	7.36E-68	1.52E-66
E2P69_RS20495	type III secretion system protein SctP	-1.492414636	4.23E-288	9.74E-286
E2P69_RS18105	S8 family serine peptidase	-1.491851137	1.07E-242	1.64E-240
E2P69_RS20535	CesT family type III secretion system chaperone	-1.489061358	2.50E-304	7.41E-302
E2P69_RS20470	type III secretion inner membrane ring lipoprotein SctJ	-1.488001547	0	0
E2P69_RS18095	S8 family serine peptidase	-1.486965649	2.57E-303	7.10E-301
E2P69_RS18250	chemotaxis protein CheA	-1.485433553	5.19E-60	9.15E-59
E2P69_RS17170	flagellar export chaperone FliS	-1.484256142	4.30E-95	1.36E-93
E2P69_RS17610	efflux transporter outer membrane subunit	-1.474878393	8.60E-53	1.31E-51
E2P69_RS20465	type III secretion protein HrpB4	-1.474506343	2.11E-296	5.14E-294
E2P69_RS19870	hypothetical protein	-1.471418188	1.21E-83	3.41E-82
E2P69_RS18265	EAL domain-containing response regulator	-1.462869737	1.80E-21	1.17E-20
E2P69_RS20520	hypothetical protein	-1.450578139	0	0
E2P69_RS20830	transporter	-1.44273196	4.04E-09	1.40E-08
E2P69_RS20505	type III secretion system export apparatus subunit SctR	-1.440429833	1.24E-152	8.42E-151
E2P69_RS05385	glucuronate isomerase	-1.437963104	1.11E-136	5.62E-135
E2P69_RS20410	lytic transglycosylase domain-containing protein	-1.42657542	1.44E-152	9.64E-151
E2P69_RS05380	endo-1,4-beta-xylanase	-1.419185153	5.81E-160	4.30E-158
E2P69_RS13540	protocatechuate 3,4-dioxygenase subunit alpha	-1.4128829	2.06E-103	7.05E-102
E2P69_RS11735	helix-turn-helix transcriptional regulator hrpX	-1.410725967	3.83E-170	3.05E-168
E2P69_RS15920	hypothetical protein	-1.407099948	8.68E-28	7.09E-27
E2P69_RS20550	HPr kinase	-1.404803811	3.42E-142	1.84E-140
E2P69_RS20515	hypothetical protein	-1.402691853	2.98E-278	5.62E-276
E2P69_RS13235	sulfotransferase domain-containing protein	-1.397929075	4.96E-140	2.57E-138
E2P69_RS11740	response regulator transcription factor-hrpG	-1.388387165	7.62E-122	3.06E-120
E2P69_RS20815	hypothetical protein	-1.38816329	1.77E-10	6.66E-10
E2P69_RS20485	type III secretion system export apparatus subunit SctU	-1.38256246	1.03E-178	8.89E-177
E2P69_RS17290	hypothetical protein	-1.377925767	1.36E-09	4.86E-09
E2P69_RS17595	autotransporter-associated beta strand repeat-containing protein	-1.367518912	0	0
E2P69_RS20490	FHIPEP family type III secretion protein	-1.351509759	1.49E-241	2.21E-239
E2P69_RS20555	HpaF protein	-1.333776479	6.02E-205	6.93E-203
E2P69_RS11440	DegQ family serine endoprotease	-1.329283209	2.65E-219	3.32E-217
E2P69_RS22045	flagellar biosynthesis protein FlhA	-1.324119648	4.71E-50	6.79E-49
E2P69_RS17075	flagellar M-ring protein FliF	-1.312126617	1.38E-97	4.45E-96
E2P69_RS17365	hypothetical protein (Type III effector)	-1.308845259	1.27E-281	2.78E-279

E2P69_RS20510	type III secretion system export apparatus subunit SctS	-1.306714915	5.33E-145	3.20E-143
E2P69_RS22515	response regulator	-1.292222448	8.29E-54	1.30E-52
E2P69_RS13545	LysR family transcriptional regulator	-1.291998392	2.75E-117	1.09E-115
E2P69_RS20460	type III secretion system stator protein SctL	-1.290058996	2.70E-131	1.21E-129
E2P69_RS17705	outer protein P	-1.28515031	3.96E-209	4.68E-207
E2P69_RS22950	outer protein P	-1.273184576	2.97E-124	1.26E-122
E2P69_RS12235	hypothetical protein	-1.272508916	5.47E-179	4.82E-177
E2P69_RS08360	DoxX family protein	-1.270029949	1.20E-15	6.12E-15
E2P69_RS20455	type III secretion system ATPase SctN	-1.269054948	1.74E-227	2.40E-225
E2P69_RS22530	ParA family protein	-1.265427165	4.60E-19	2.71E-18
E2P69_RS22945	hypothetical protein	-1.257395439	1.44E-135	7.02E-134
E2P69_RS02240	MBL fold metallo-hydrolase	-1.245443267	1.51E-124	6.47E-123
E2P69_RS20440	type III secretion system outer membrane ring subunit SctC	-1.245335194	1.23E-186	1.18E-184
E2P69_RS17615	CusA/CzcA family heavy metal efflux RND transporter	-1.234722263	1.16E-84	3.31E-83
E2P69_RS16395	hypothetical protein	-1.222995604	3.19E-110	1.17E-108
E2P69_RS17925	serine/threonine protein kinase	-1.216616901	7.17E-198	7.82E-196
E2P69_RS20450	type III secretion protein HrpB7	-1.214635183	1.97E-133	9.19E-132
E2P69_RS13535	gallate dioxygenase	-1.207841745	3.67E-147	2.30E-145
E2P69_RS09155	MCP four helix bundle domain-containing protein	-1.198628712	1.82E-29	1.57E-28
E2P69_RS20525	hypothetical protein	-1.191928945	1.27E-113	4.78E-112
E2P69_RS16300	FAD/NAD(P)-binding protein	-1.184829981	3.17E-90	9.67E-89
E2P69_RS22980	hypothetical protein	-1.183918884	8.06E-144	4.57E-142
E2P69_RS22970	hypothetical protein	-1.170168003	9.84E-42	1.17E-40
E2P69_RS20445	type III secretion system export apparatus subunit SctT	-1.167255669	3.39E-159	2.46E-157
E2P69_RS20165	hypothetical protein	-1.164599982	2.57E-29	2.20E-28
E2P69_RS20845	MCP four helix bundle domain-containing protein	-1.149558365	3.62E-71	8.02E-70
E2P69_RS04870	hypothetical protein	-1.147858745	6.26E-44	7.85E-43
E2P69_RS22965	hypothetical protein	-1.143919205	5.27E-147	3.26E-145
E2P69_RS10580	hypothetical protein	-1.138328673	3.32E-13	1.48E-12
E2P69_RS22015	flagellar biosynthetic protein FliR	-1.136438618	1.40E-27	1.14E-26
E2P69_RS11900	SPFH domain-containing protein	-1.134939212	1.53E-143	8.57E-142
E2P69_RS00225	glycoside hydrolase family 5 protein	-1.132879932	8.43E-91	2.61E-89
E2P69_RS15610	hypothetical protein	-1.132238408	1.03E-187	1.01E-185
E2P69_RS22055	MinD/ParA family protein	-1.128317841	1.10E-34	1.09E-33
E2P69_RS14790	hypothetical protein	-1.12041609	1.48E-68	3.13E-67
E2P69_RS18200	hypothetical protein	-1.10926808	1.83E-05	4.59E-05
E2P69_RS17910	TetR/AcrR family transcriptional regulator	-1.108784247	1.61E-154	1.13E-152
E2P69_RS07020	hypothetical protein	-1.106407527	0.005206355	0.009655295
E2P69_RS18110	hypothetical protein	-1.105295491	9.24E-51	1.36E-49
E2P69_RS04055	phage virion morphogenesis protein	-1.104171591	7.79E-52	1.17E-50
E2P69_RS01455	hypothetical protein	-1.101971977	1.09E-179	9.84E-178
E2P69_RS00885	amidohydrolase	-1.097022551	2.91E-120	1.16E-118
E2P69_RS17915	hypothetical protein	-1.093245544	4.68E-144	2.69E-142
E2P69_RS03430	thioredoxin family protein	-1.092423544	1.81E-135	8.71E-134
E2P69_RS05375	hypothetical protein	-1.087401557	3.32E-48	4.61E-47

E2P69_RS17230	flagellar basal body rod modification protein FlgD	-1.086568002	1.45E-31	1.31E-30
E2P69_RS13970	C40 family peptidase	-1.08581477	5.68E-30	5.00E-29
E2P69_RS18550	hypothetical protein	-1.084257613	2.42E-77	5.91E-76
E2P69_RS13380	TauD/TfdA family dioxygenase	-1.081981051	2.90E-65	5.64E-64
E2P69_RS17565	stress-induced protein	-1.06898123	7.41E-20	4.53E-19
E2P69_RS01410	GTP-binding protein	-1.067819476	1.16E-50	1.69E-49
E2P69_RS15620	DUF3182 family protein	-1.06357134	1.16E-135	5.74E-134
E2P69_RS01490	hypothetical protein	-1.060247219	6.08E-107	2.17E-105
E2P69_RS19335	conjugal transfer transcriptional regulator TraJ	-1.051663896	3.39E-05	8.27E-05
E2P69_RS18345	ankyrin repeat domain-containing protein	-1.047851218	2.61E-58	4.50E-57
E2P69_RS02000	helix-hairpin-helix domain-containing protein	-1.04413536	9.88E-23	6.68E-22
E2P69_RS18570	hypothetical protein	-1.040026335	1.58E-07	4.80E-07
E2P69_RS19420	P-type conjugative transfer protein TrbJ	-1.03576842	6.37E-137	3.26E-135
E2P69_RS20905	MCP four helix bundle domain-containing protein	-1.032628149	4.03E-22	2.69E-21
E2P69_RS04715	hypothetical protein	-1.03225822	3.75E-82	1.01E-80
E2P69_RS18710	hypothetical protein	-1.030994683	0.006424717	0.011730984
E2P69_RS16205	thioredoxin family protein	-1.022406235	1.67E-73	3.86E-72
E2P69_RS00340	avirulence protein	-1.009541433	1.48E-133	6.97E-132
E2P69_RS18575	enoyl-CoA hydratase/isomerase family protein	-1.007156108	1.93E-45	2.53E-44
E2P69_RS02735	hypothetical protein	-1.003230157	7.58E-89	2.23E-87
E2P69_RS11450	RNA polymerase sigma factor RpoE	-1.000313236	1.89E-102	6.37E-101
E2P69_RS20160	hypothetical protein	-1.000130596	2.35E-17	1.28E-16
E2P69_RS08355	amidohydrolase family protein	-0.995150453	3.31E-14	1.56E-13
E2P69_RS11290	thiolase family protein	-0.992391425	4.00E-71	8.82E-70
E2P69_RS12985	F-box protein	-0.991361613	8.33E-98	2.72E-96
E2P69_RS18205	cell envelope integrity protein CreD	-0.990500235	5.77E-25	4.19E-24
E2P69_RS01415	hypothetical protein	-0.984736638	4.03E-113	1.51E-111
E2P69_RS09870	3-isopropylmalate dehydratase small subunit	-0.974557629	2.62E-31	2.37E-30
E2P69_RS08130	succinate dehydrogenase, cytochrome b556 subunit	-0.974133605	1.12E-67	2.29E-66
E2P69_RS04050	type III secretion system YopJ family effector AvrXv4	-0.97394433	7.67E-79	1.92E-77
E2P69_RS02665	sigma-70 family RNA polymerase sigma factor	-0.97148555	3.37E-08	1.08E-07
E2P69_RS22510	chemotaxis protein CheA	-0.966649762	4.33E-44	5.46E-43
E2P69_RS06515	diguanylate cyclase	-0.966425221	4.04E-16	2.10E-15
E2P69_RS05600	hypothetical protein	-0.965894839	3.53E-122	1.43E-120
E2P69_RS10085	rubredoxin	-0.96098979	1.29E-30	1.15E-29
E2P69_RS20435	IS1595 family transposase	-0.960134954	2.26E-71	5.03E-70
E2P69_RS19935	hypothetical protein	-0.956697681	5.44E-45	7.07E-44
E2P69_RS17040	flagellar motor switch protein FliM	-0.956174338	1.73E-39	1.95E-38
E2P69_RS00865	phenylalanine 4-monooxygenase	-0.95535375	7.35E-30	6.43E-29
E2P69_RS00280	penicillin acylase family protein	-0.955221998	1.81E-129	8.07E-128
E2P69_RS18210	C1 family peptidase	-0.953575779	1.96E-65	3.87E-64
E2P69_RS20545	hypothetical protein	-0.950247308	2.16E-131	9.82E-130

E2P69_RS14760	hypothetical protein	-0.949711303	7.88E-74	1.85E-72
E2P69_RS00235	glycoside hydrolase family 5 protein	-0.946412227	6.53E-66	1.30E-64
E2P69_RS15045	YadA-like family protein	-0.943740428	0.020754919	0.034054506
E2P69_RS02500	serine hydrolase	-0.940504856	1.83E-75	4.38E-74
E2P69_RS08125	succinate dehydrogenase, hydrophobic membrane anchor protein	-0.937532916	4.40E-49	6.25E-48
E2P69_RS16295	VirK family protein	-0.936454277	8.60E-53	1.31E-51
E2P69_RS22525	chemotaxis protein CheW	-0.932316137	9.14E-15	4.45E-14
E2P69_RS03165	type III PLP-dependent enzyme	-0.929314376	0.000640716	0.001332575
E2P69_RS11910	hypothetical protein	-0.920522671	4.37E-40	4.97E-39
E2P69_RS15430	zinc-finger domain-containing protein	-0.92050197	1.12E-29	9.73E-29
E2P69_RS02645	leucine-rich repeat domain-containing protein	-0.917295928	1.35E-79	3.44E-78
E2P69_RS22660	transposase	-0.915062923	6.14E-10	2.24E-09
E2P69_RS10575	SDR family oxidoreductase	-0.913054032	2.42E-11	9.61E-11
E2P69_RS11555	VOC family protein	-0.912111255	4.18E-49	5.96E-48
E2P69_RS20170	hypothetical protein	-0.909901944	2.67E-05	6.61E-05
E2P69_RS02615	type III secretion system effector protein XopK	-0.906642255	2.99E-104	1.03E-102
E2P69_RS11035	DUF502 domain-containing protein	-0.902716462	1.78E-56	2.95E-55
E2P69_RS09330	TonB-dependent receptor	-0.901025667	3.47E-66	6.94E-65
E2P69_RS15615	alpha/beta fold hydrolase	-0.897276286	1.54E-65	3.06E-64
E2P69_RS02110	ribosome-associated translation inhibitor RaiA	-0.896449235	3.40E-48	4.71E-47
E2P69_RS20890	flagellar brake protein	-0.895993548	3.15E-09	1.10E-08
E2P69_RS13225	DUF3228 family protein	-0.887961004	2.54E-12	1.09E-11
E2P69_RS12070	hypothetical protein	-0.885496534	4.90E-34	4.72E-33
E2P69_RS10450	prephenate dehydrogenase	-0.884158951	2.82E-70	6.12E-69
E2P69_RS17195	flagellar assembly peptidoglycan hydrolase FlgJ	-0.8792555	1.47E-26	1.14E-25
E2P69_RS12300	GGDEF domain-containing protein	-0.878024221	0.000151171	0.000339827
E2P69_RS22765	IS3 family transposase	-0.877905018	1.08E-47	1.49E-46
E2P69_RS13145	DUF721 domain-containing protein	-0.875365762	2.48E-44	3.16E-43
E2P69_RS17160	PilZ domain-containing protein	-0.873937125	3.47E-20	2.16E-19
E2P69_RS13870	23S rRNA (pseudouridine(1915)-N(3))-methyltransferase RlmH	-0.870166666	2.71E-13	1.21E-12
E2P69_RS18350	DUF2974 domain-containing protein	-0.866041769	1.12E-83	3.17E-82
E2P69_RS11445	hypothetical protein	-0.865778716	6.60E-16	3.40E-15
E2P69_RS13190	S8/S53 family peptidase	-0.865511378	4.83E-77	1.17E-75
E2P69_RS08725	cytochrome ubiquinol oxidase subunit I	-0.864809792	2.41E-16	1.26E-15
E2P69_RS20740	uroporphyrinogen-III C-methyltransferase	-0.860887842	2.52E-29	2.16E-28
E2P69_RS22895	hypothetical protein	-0.855675359	5.57E-07	1.59E-06
E2P69_RS14725	hypothetical protein	-0.851498068	3.69E-32	3.37E-31
E2P69_RS18565	endonuclease III	-0.850286775	5.30E-23	3.62E-22
E2P69_RS07920	response regulator	-0.849134936	7.04E-49	9.93E-48
E2P69_RS21485	hypothetical protein	-0.845933972	2.59E-42	3.13E-41
E2P69_RS17205	flagellar basal body L-ring protein FlgH	-0.844330513	2.44E-11	9.67E-11
E2P69_RS12375	S9 family peptidase	-0.84369313	2.72E-68	5.69E-67
E2P69_RS08195	c-type cytochrome	-0.842840201	2.95E-36	3.00E-35
E2P69_RS11010	polyhydroxyalkanoate depolymerase	-0.84178256	1.78E-81	4.70E-80

E2P69_RS08135	DUF1674 domain-containing protein	-0.840730634	4.12E-46	5.52E-45
E2P69_RS10375	OmpW family protein	-0.83768164	1.10E-09	3.94E-09
E2P69_RS05475	hypothetical protein	-0.835616007	1.19E-09	4.24E-09
E2P69_RS09150	purine-binding chemotaxis protein CheW	-0.835115992	3.15E-08	1.01E-07
E2P69_RS17585	DUF1349 domain-containing protein	-0.829686285	1.82E-21	1.18E-20
E2P69_RS14015	M13 family metallopeptidase	-0.829506276	6.24E-55	9.94E-54
E2P69_RS21680	MFS transporter	-0.826130694	2.13E-44	2.73E-43
E2P69_RS02215	lipase	-0.82541226	2.94E-52	4.48E-51
E2P69_RS08140	folate-binding protein YgfZ	-0.825208508	5.64E-30	4.97E-29
E2P69_RS06205	S8/S53 family peptidase	-0.820405674	9.12E-15	4.45E-14
E2P69_RS00500	SRPBCC domain-containing protein	-0.816785529	9.54E-29	8.03E-28
E2P69_RS07935	FKBP-type peptidyl-prolyl cis-trans isomerase	-0.810384283	1.28E-06	3.59E-06
E2P69_RS10725	MarR family transcriptional regulator	-0.808127294	4.68E-60	8.29E-59
E2P69_RS02075	BolA/IbaG family iron-sulfur metabolism protein	-0.806574908	3.21E-25	2.36E-24
E2P69_RS01955	Fe2+-dependent dioxygenase	-0.803778151	0.017147997	0.028704708
E2P69_RS16590	YbaB/EbfC family nucleoid-associated protein	-0.803362884	2.29E-47	3.15E-46
E2P69_RS09865	3-isopropylmalate dehydratase large subunit	-0.802796401	2.41E-48	3.36E-47
E2P69_RS17190	flagellar hook-associated protein FlgK	-0.802399086	9.36E-28	7.64E-27
E2P69_RS21585	protocatechuate 3,4-dioxygenase subunit beta	-0.797118457	6.56E-23	4.46E-22
E2P69_RS17165	hypothetical protein	-0.795386886	1.07E-16	5.69E-16
E2P69_RS14785	IS3 family transposase	-0.794519292	0.000762742	0.00157608
E2P69_RS11915	hypothetical protein	-0.790927837	2.37E-20	1.49E-19
E2P69_RS22060	RNA polymerase sigma factor FliA	-0.789369299	1.59E-10	5.98E-10
E2P69_RS04985	fumarylacetoacetate hydrolase family protein	-0.787592688	1.66E-16	8.74E-16
E2P69_RS22915	hypothetical protein	-0.786678609	7.65E-15	3.76E-14
E2P69_RS15945	lipase	-0.786048791	5.68E-103	1.93E-101
E2P69_RS01765	hypothetical protein	-0.783164004	1.34E-19	8.12E-19
E2P69_RS02800	pyrroloquinoline quinone biosynthesis protein PqqB	-0.782112356	6.31E-61	1.13E-59
E2P69_RS12250	alpha,alpha-trehalase TreA	-0.778687317	1.11E-37	1.18E-36
E2P69_RS04410	hypothetical protein	-0.77428011	4.20E-07	1.21E-06
E2P69_RS18915	hypothetical protein	-0.773646842	7.29E-57	1.22E-55
E2P69_RS11550	tryptophan 2,3-dioxygenase	-0.773125065	4.83E-42	5.82E-41
E2P69_RS13315	carbohydrate-binding protein	-0.772719764	8.77E-76	2.11E-74
E2P69_RS17245	chemotaxis protein CheV	-0.772167201	2.47E-34	2.40E-33
E2P69_RS13375	ABC transporter substrate-binding protein	-0.766139115	1.06E-26	8.32E-26
E2P69_RS16525	MEKHLA domain-containing protein	-0.762975138	6.41E-62	1.18E-60
E2P69_RS03800	peptide deformylase	-0.762932867	3.65E-50	5.29E-49
E2P69_RS01795	hypothetical protein	-0.762506	0.00068661	0.001423024
E2P69_RS14845	nucleoside hydrolase	-0.761076817	1.55E-54	2.46E-53
E2P69_RS01550	hypothetical protein	-0.760185854	5.84E-34	5.59E-33
E2P69_RS14125	SCO family protein	-0.759842953	6.93E-17	3.72E-16
E2P69_RS08120	succinate dehydrogenase flavoprotein subunit	-0.757864333	6.27E-72	1.41E-70
E2P69_RS04645	methyltransferase	-0.753430562	1.70E-16	8.96E-16

E2P69_RS17485	exodeoxyribonuclease III	-0.750839939	2.12E-32	1.95E-31
E2P69_RS02630	RebB family R body protein	-0.749219218	1.13E-20	7.16E-20
E2P69_RS04375	hypothetical protein	-0.748449262	1.81E-39	2.03E-38
E2P69_RS22065	chemotaxis response regulator CheY	-0.748006421	4.55E-12	1.91E-11
E2P69_RS08115	hypothetical protein	-0.745263	7.50E-14	3.47E-13
E2P69_RS20745	cysteine synthase A	-0.74481459	9.11E-47	1.24E-45
E2P69_RS03250	GIY-YIG nuclease family protein	-0.743272247	4.44E-06	1.19E-05
E2P69_RS11730	ATP-binding protein	-0.742656466	8.12E-29	6.85E-28
E2P69_RS13070	division/cell wall cluster transcriptional repressor MraZ	-0.742418908	8.37E-35	8.31E-34
E2P69_RS04575	flagellar motor stator protein MotA	-0.741726879	2.13E-18	1.21E-17
E2P69_RS00645	DUF2141 domain-containing protein	-0.737162068	1.48E-36	1.51E-35
E2P69_RS20540	hypothetical protein- next to T3SS	-0.732288428	3.94E-78	9.72E-77
E2P69_RS03135	4-hydroxy-2-oxovalerate aldolase	-0.731054315	0.029428601	0.046821311
E2P69_RS12610	bifunctional nicotinamide-nucleotide adenylyltransferase/Nudix hydroxylase	-0.731042692	1.45E-43	1.80E-42
E2P69_RS21590	3-oxoadipyl-CoA thiolase	-0.729899541	8.62E-17	4.61E-16
E2P69_RS08350	MFS transporter	-0.726315594	6.90E-08	2.15E-07
E2P69_RS10150	PilZ domain-containing protein	-0.726085225	0.001249384	0.002523743
E2P69_RS10645	hypothetical protein	-0.724187868	6.04E-14	2.81E-13
E2P69_RS07890	4a-hydroxytetrahydrobiopterin dehydratase	-0.722268414	3.37E-14	1.59E-13
E2P69_RS15935	catalase	-0.72031075	1.02E-13	4.69E-13
E2P69_RS01775	hypothetical protein	-0.718579547	0.000122905	0.000279777
E2P69_RS19315	hypothetical protein	-0.712406912	6.91E-31	6.22E-30
E2P69_RS11865	hypothetical protein	-0.710961478	6.71E-55	1.07E-53
E2P69_RS08200	cytochrome c	-0.709152122	4.03E-15	2.01E-14
E2P69_RS09975	adenylate kinase	-0.707793678	1.48E-40	1.72E-39
E2P69_RS21230	ferrous iron transport protein A	-0.706758441	1.04E-10	3.95E-10
E2P69_RS10570	efflux RND transporter periplasmic adaptor subunit	-0.705457864	1.58E-09	5.61E-09
E2P69_RS15875	hypothetical protein	-0.703732555	4.51E-19	2.67E-18
E2P69_RS20730	hypothetical protein	-0.700288018	4.63E-05	0.000110966
E2P69_RS14755	hypothetical protein	-0.699748457	1.17E-34	1.15E-33
E2P69_RS22070	protein phosphatase CheZ	-0.695939907	4.80E-09	1.65E-08
E2P69_RS13080	cell division protein FtsL	-0.695060411	1.39E-07	4.24E-07
E2P69_RS02810	pyrroloquinoline quinone biosynthesis peptide chaperone PqqD	-0.690787452	1.81E-20	1.14E-19
E2P69_RS12240	magnesium transporter CorA family protein	-0.68961902	1.30E-18	7.50E-18
E2P69_RS11070	VOC family protein	-0.689597485	4.18E-07	1.21E-06
E2P69_RS15250	hypothetical protein	-0.687223759	0.028012028	0.044877738
E2P69_RS00695	sugar kinase	-0.687218486	2.75E-11	1.09E-10
E2P69_RS05570	Sec-independent protein translocase subunit TatA	-0.686911568	4.72E-23	3.23E-22
E2P69_RS02225	trypsin-like peptidase domain-containing protein	-0.686415971	2.55E-64	4.82E-63
E2P69_RS17670	hypothetical protein	-0.686002798	2.00E-43	2.47E-42
E2P69_RS02235	hypothetical protein	-0.68493091	7.20E-12	2.97E-11
E2P69_RS10445	hypothetical protein	-0.684405559	3.09E-39	3.43E-38
E2P69_RS17930	hypothetical protein	-0.678232532	5.58E-08	1.75E-07
E2P69_RS02805	pyrroloquinoline-quinone synthase	-0.677539401	2.43E-28	2.02E-27

	PqqC			
E2P69_RS11040	queuosine precursor transporter	-0.676841359	3.17E-40	3.62E-39
E2P69_RS13125	cell division protein FtsQ/DivIB	-0.675762627	4.32E-11	1.69E-10
E2P69_RS15600	slipin family protein	-0.674355865	6.79E-16	3.49E-15
E2P69_RS03260	DUF3014 domain-containing protein	-0.673526667	1.90E-34	1.85E-33
E2P69_RS16200	MarR family transcriptional regulator	-0.673334584	3.13E-25	2.30E-24
E2P69_RS07265	MFS transporter	-0.669905645	6.20E-41	7.26E-40
E2P69_RS13550	AMP-binding protein	-0.669712357	4.63E-08	1.46E-07
E2P69_RS22650	hypothetical protein	-0.666475698	1.44E-21	9.40E-21
E2P69_RS03175	hypothetical protein	-0.666399411	3.36E-29	2.86E-28
E2P69_RS07235	DUF47 family protein	-0.662343534	3.66E-09	1.27E-08
E2P69_RS13875	TonB family protein	-0.661346734	1.81E-14	8.67E-14
E2P69_RS14720	hypothetical protein	-0.659099816	2.42E-14	1.15E-13
E2P69_RS20735	LysR family transcriptional regulator	-0.658502393	9.14E-13	3.99E-12
E2P69_RS12065	hypothetical protein	-0.656084193	5.57E-42	6.69E-41
E2P69_RS05000	DUF4398 domain-containing protein	-0.656079172	7.70E-18	4.32E-17
E2P69_RS18405	single-stranded DNA-binding protein	-0.655355023	6.67E-43	8.12E-42
E2P69_RS18955	aspartate 1-decarboxylase	-0.654210971	1.34E-29	1.16E-28
E2P69_RS08330	Nramp family divalent metal transporter	-0.654192662	1.42E-37	1.50E-36
E2P69_RS03290	TonB-dependent receptor	-0.654152727	5.72E-52	8.62E-51
E2P69_RS11545	FAD-dependent monooxygenase	-0.654032866	3.82E-26	2.90E-25
E2P69_RS17945	type I restriction-modification system endonuclease	-0.653089448	1.58E-34	1.54E-33
E2P69_RS22695	chemotaxis protein CheW	-0.652947038	7.61E-06	2.00E-05
E2P69_RS20820	cache domain-containing protein	-0.652894965	1.54E-08	5.08E-08
E2P69_RS08110	succinate dehydrogenase iron-sulfur subunit	-0.651662604	1.23E-33	1.16E-32
E2P69_RS02605	glycoside hydrolase family 92 protein	-0.651356301	3.55E-20	2.20E-19
E2P69_RS15825	hypothetical protein	-0.650312181	2.99E-14	1.41E-13
E2P69_RS01685	hypothetical protein	-0.649008725	5.56E-52	8.41E-51
E2P69_RS01680	efflux transporter outer membrane subunit	-0.64867062	7.88E-12	3.25E-11
E2P69_RS02610	beta-galactosidase	-0.6470693	1.68E-20	1.06E-19
E2P69_RS09875	3-isopropylmalate dehydrogenase	-0.64656596	1.48E-24	1.06E-23
E2P69_RS13230	hypothetical protein	-0.645443157	3.64E-27	2.93E-26
E2P69_RS17380	fluoride efflux transporter CrcB	-0.644878072	5.09E-24	3.60E-23
E2P69_RS12230	D-serine/D-alanine/glycine transporter	-0.643347329	1.59E-24	1.14E-23
E2P69_RS11000	CopD family protein	-0.641540707	7.98E-12	3.28E-11
E2P69_RS13220	DUF2058 domain-containing protein	-0.640889845	2.17E-19	1.31E-18
E2P69_RS16890	hypothetical protein	-0.638815099	3.48E-38	3.76E-37
E2P69_RS18305	hypothetical protein	-0.63779827	4.10E-20	2.54E-19
E2P69_RS04490	aldo/keto reductase	-0.636400873	9.89E-43	1.20E-41
E2P69_RS08480	bifunctional methylenetetrahydrofolate dehydrogenase/methenyltetrahydrofolate cyclohydrolase FolD	-0.636310753	3.96E-23	2.73E-22
E2P69_RS13800	DUF378 domain-containing protein	-0.635994816	6.03E-22	3.99E-21
E2P69_RS11725	hypothetical protein	-0.635740421	1.76E-28	1.47E-27
E2P69_RS16770	isocitrate dehydrogenase	-0.635402569	6.19E-40	7.01E-39
E2P69_RS22925	hypothetical protein	-0.63487169	6.90E-17	3.71E-16
E2P69_RS19085	CPBP family intramembrane metalloprotease	-0.634645027	6.30E-14	2.92E-13

E2P69_RS02815	pyrroloquinoline quinone biosynthesis protein PqqE	-0.632115905	8.59E-26	6.43E-25
E2P69_RS17490	membrane protein	-0.631470509	2.22E-09	7.80E-09
E2P69_RS05730	AAA family ATPase	-0.631412343	1.47E-06	4.09E-06
E2P69_RS13810	hypothetical protein	-0.631229414	3.28E-05	8.02E-05
E2P69_RS02210	hypothetical protein	-0.630865033	3.53E-11	1.39E-10
E2P69_RS07005	hypothetical protein	-0.629256305	0.027253431	0.043746984
E2P69_RS18310	pirin family protein	-0.629217897	2.09E-08	6.80E-08
E2P69_RS20270	STAS domain-containing protein	-0.629002536	5.79E-27	4.60E-26
E2P69_RS21155	acireductone dioxygenase	-0.628154995	5.27E-27	4.20E-26
E2P69_RS05235	GAF domain-containing sensor histidine kinase	-0.626689211	2.40E-24	1.72E-23
E2P69_RS14795	nuclear transport factor 2 family protein	-0.626481563	6.57E-19	3.84E-18
E2P69_RS14120	phosphatidylserine decarboxylase	-0.624712557	2.66E-11	1.05E-10
E2P69_RS04650	rRNA pseudouridine synthase	-0.624629984	2.26E-08	7.30E-08
E2P69_RS07885	NfuA family Fe-S biogenesis protein	-0.62090699	2.79E-35	2.80E-34
E2P69_RS03575	VOC family protein	-0.620721116	9.14E-18	5.11E-17
E2P69_RS07925	sulfurtransferase	-0.620719027	1.80E-11	7.24E-11
E2P69_RS22150	tyrosine--tRNA ligase	-0.620164988	3.60E-23	2.49E-22
E2P69_RS16355	GGDEF domain-containing protein	-0.620071224	1.80E-09	6.34E-09
E2P69_RS21595	CoA-transferase subunit beta	-0.619683497	2.93E-09	1.02E-08
E2P69_RS13120	D-alanine--D-alanine ligase	-0.618149099	1.83E-13	8.31E-13
E2P69_RS20680	sulfate adenylyltransferase subunit CysD	-0.617693449	1.70E-22	1.15E-21
E2P69_RS01315	GGDEF domain-containing protein	-0.615597034	3.96E-24	2.81E-23
E2P69_RS16915	glycine C-acetyltransferase	-0.615099514	1.79E-14	8.58E-14
E2P69_RS09970	6-phosphofructokinase	-0.614367064	7.06E-21	4.48E-20
E2P69_RS03320	methyl-accepting chemotaxis protein	-0.614035977	3.77E-09	1.31E-08
E2P69_RS09925	phosphomethylpyrimidine synthase ThiC	-0.612832788	7.94E-34	7.58E-33
E2P69_RS01345	methylcrotonoyl-CoA carboxylase	-0.612441353	2.08E-13	9.41E-13
E2P69_RS10145	Xaa-Pro dipeptidase	-0.612171524	1.13E-16	6.02E-16
E2P69_RS10560	SDR family oxidoreductase	-0.611835883	0.000115129	0.000263234
E2P69_RS09645	ribonucleoside-diphosphate reductase subunit alpha	-0.610679099	5.96E-54	9.36E-53
E2P69_RS00625	hypothetical protein	-0.610206244	1.29E-18	7.46E-18
E2P69_RS07930	hypothetical protein	-0.609584811	1.92E-05	4.81E-05
E2P69_RS15990	(2Fe-2S)-binding protein	-0.609015372	6.03E-12	2.50E-11
E2P69_RS21605	aromatic ring-hydroxylating dioxygenase subunit alpha	-0.608779951	4.69E-18	2.64E-17
E2P69_RS01815	OsmC family protein	-0.607563683	2.64E-31	2.38E-30
E2P69_RS19065	DUF4190 domain-containing protein	-0.606895956	5.37E-19	3.16E-18
E2P69_RS18865	imidazolonepropionase	-0.603051517	0.000409325	0.000872791
E2P69_RS03170	hypothetical protein	-0.602908138	1.34E-23	9.36E-23
E2P69_RS08985	adenine phosphoribosyltransferase	-0.600031141	3.62E-19	2.15E-18
E2P69_RS14615	YchJ family protein	-0.599860327	0.00047583	0.00100426
E2P69_RS13675	pseudouridine synthase	-0.599338747	1.09E-14	5.28E-14
E2P69_RS04160	ParD-like family protein	-0.5935443	4.03E-16	2.10E-15
E2P69_RS13370	ABC transporter ATP-binding protein	-0.593164031	3.86E-19	2.29E-18
E2P69_RS13860	ribosome silencing factor	-0.592263165	7.90E-23	5.36E-22
E2P69_RS13705	serine hydrolase	-0.59224235	1.01E-11	4.14E-11
E2P69_RS11455	enoyl-CoA hydratase/isomerase family	-0.592063881	3.83E-38	4.12E-37

	protein			
E2P69_RS03640	hypothetical protein	-0.590579377	1.53E-23	1.06E-22
E2P69_RS06715	LysR family transcriptional regulator	-0.588518413	8.22E-06	2.15E-05
E2P69_RS22690	hypothetical protein	-0.58668313	3.56E-08	1.13E-07
E2P69_RS16290	efflux transporter outer membrane subunit	-0.584977897	1.14E-37	1.20E-36
E2P69_RS05575	twin-arginine translocase subunit TatB	-0.584047544	3.79E-18	2.15E-17
E2P69_RS20085	bifunctional phosphoribosylaminoimidazolecarboxamide formyltransferase/IMP cyclohydrolase	-0.583252496	5.09E-22	3.38E-21
E2P69_RS03465	tRNA (adenosine(37)-N6)-threonylcarbamoyltransferase complex transferase subunit TsaD	-0.582255268	1.56E-18	8.99E-18
E2P69_RS12035	hypothetical protein	-0.580495333	9.67E-33	9.00E-32

Table S1 (D) Complete list of upregulated genes in the mutant strain post 16 hours of growth in XVM2 medium

Locus tag	Annotation	log2FC	pvalue	padj
E2P69_RS22570	YadA-like family protein	3.411900985	0.007616805	0.013690422
E2P69_RS11815	GTPase ObgE	2.418813944	0	0
E2P69_RS14460	GspH/FimT family pseudopilin	2.345525609	2.37E-40	2.72E-39
E2P69_RS19750	methyltransferase domain-containing protein	2.183829764	3.06E-281	6.34E-279
E2P69_RS07775	YeeE/YedE family protein	2.132427737	2.13E-46	2.87E-45
E2P69_RS19745	flippase-like domain-containing protein	2.102193141	0	0
E2P69_RS19755	hypothetical protein	2.019362663	0	0
E2P69_RS17560	uroporphyrinogen-III C-methyltransferase	2.001105515	6.35E-80	1.62E-78
E2P69_RS12410	tyrosine recombinase XerC	1.865523642	1.07E-51	1.60E-50
E2P69_RS04270	DUF2894 domain-containing protein	1.859030587	1.43E-19	8.63E-19
E2P69_RS04275	OmpA family protein	1.820658793	1.70E-09	6.04E-09
E2P69_RS10215	type B 50S ribosomal protein L31	1.81632741	8.63E-230	1.23E-227
E2P69_RS04280	DUF802 domain-containing protein	1.792787255	1.21E-66	2.44E-65
E2P69_RS21095	trp operon repressor	1.753585759	1.48E-41	1.76E-40
E2P69_RS22105	DNA-directed RNA polymerase subunit beta	1.749746899	0	0
E2P69_RS06880	hypothetical protein	1.749126543	0.007319812	0.01320818
E2P69_RS22100	DNA-directed RNA polymerase subunit beta'	1.735925585	0	0
E2P69_RS09760	TonB-dependent receptor	1.712235952	3.51E-143	1.94E-141
E2P69_RS06765	C40 family peptidase	1.710473189	1.57E-05	3.97E-05
E2P69_RS19905	TonB-dependent receptor	1.695446493	0	0
E2P69_RS11805	murein biosynthesis integral membrane protein MurJ	1.687155166	3.39E-129	1.49E-127
E2P69_RS11600	tRNA (guanosine(37)-N1)-methyltransferase TrmD	1.676140818	6.23E-220	8.06E-218
E2P69_RS14440	Tfp pilus assembly protein PilX	1.641319688	3.57E-247	5.91E-245
E2P69_RS21100	ATP phosphoribosyltransferase HisG	1.622204911	3.03E-72	6.91E-71
E2P69_RS17545	NAD(P)/FAD-dependent oxidoreductase	1.615280424	2.30E-150	1.49E-148
E2P69_RS07770	MBL fold metallo-hydrolase	1.613944336	9.59E-38	1.02E-36
E2P69_RS19760	pilin	1.612140765	1.48E-112	5.47E-111
E2P69_RS17550	nitrite reductase small subunit NirD	1.609038995	9.89E-27	7.75E-26
E2P69_RS21125	1-(5-phosphoribosyl)-5-[(5-phosphoribosylamino)methylideneamino]imidazole-4-carboxamide isomerase HisA	1.593666524	1.38E-82	3.75E-81
E2P69_RS17690	type VI secretion system contractile sheath small subunit	1.582424591	2.92E-05	7.19E-05
E2P69_RS07780	membrane protein	1.577533115	1.18E-19	7.20E-19
E2P69_RS17555	molybdopterin-dependent oxidoreductase	1.559343428	3.90E-105	1.37E-103
E2P69_RS12820	betaine-aldehyde dehydrogenase	1.521953838	1.01E-153	7.01E-152
E2P69_RS12405	DUF484 family protein	1.510138601	3.40E-65	6.59E-64
E2P69_RS12600	LysR family transcriptional regulator	1.490629942	3.69E-28	3.03E-27
E2P69_RS07055	AlpA family phage regulatory	1.490098392	2.93E-68	6.10E-67

	protein			
E2P69_RS06890	glycoside hydrolase family 19 protein	1.485784945	8.72E-08	2.70E-07
E2P69_RS12815	choline dehydrogenase	1.4840157	7.39E-192	7.47E-190
E2P69_RS22345	elongation factor Tu	1.482654127	6.42E-63	1.20E-61
E2P69_RS15710	RNA polymerase sigma factor RpoE	1.481335334	2.10E-40	2.43E-39
E2P69_RS22610	type VI secretion system tube protein Hcp	1.462627409	1.11E-17	6.18E-17
E2P69_RS14435	PilW family protein	1.462579704	1.46E-220	1.95E-218
E2P69_RS13430	TPM domain-containing protein	1.455812088	5.60E-56	9.13E-55
E2P69_RS03455	GatB/YqeY domain-containing protein	1.451374462	3.77E-141	1.98E-139
E2P69_RS12400	diaminopimelate epimerase	1.428151183	2.10E-106	7.45E-105
E2P69_RS21395	hypothetical protein	1.423984992	4.97E-51	7.35E-50
E2P69_RS17540	NarK/NasA family nitrate transporter	1.421537246	2.45E-90	7.52E-89
E2P69_RS14445	pilus assembly protein PilY1	1.419053949	4.51E-259	8.13E-257
E2P69_RS07050	hypothetical protein	1.413324773	4.85E-34	4.68E-33
E2P69_RS12165	S9 family peptidase	1.409658974	7.23E-85	2.08E-83
E2P69_RS17090	polysaccharide pyruvyl transferase family protein	1.390352639	1.41E-70	3.08E-69
E2P69_RS12825	choline BCCT transporter BetT	1.385927056	3.38E-164	2.59E-162
E2P69_RS21015	hypothetical protein	1.382045015	5.23E-17	2.83E-16
E2P69_RS12595	sulfite exporter TauE/SafE family protein	1.381220196	3.60E-45	4.71E-44
E2P69_RS06680	hypothetical protein	1.379831805	8.91E-45	1.15E-43
E2P69_RS01045	ammonium transporter	1.375946681	7.05E-192	7.30E-190
E2P69_RS01050	signal transduction histidine kinase	1.369619074	1.16E-69	2.51E-68
E2P69_RS04415	hypothetical protein	1.368938832	1.29E-171	1.05E-169
E2P69_RS01055	nitrogen regulation protein NR(I)	1.364824185	1.59E-49	2.27E-48
E2P69_RS06885	hypothetical protein	1.359302456	2.39E-05	5.93E-05
E2P69_RS22465	preprotein translocase subunit SecY	1.353908084	1.28E-136	6.41E-135
E2P69_RS11190	RNA 2',3'-cyclic phosphodiesterase	1.348183298	1.27E-37	1.34E-36
E2P69_RS09275	ABC transporter permease subunit	1.341133985	7.91E-41	9.23E-40
E2P69_RS08770	hypothetical protein	1.340623724	9.65E-09	3.22E-08
E2P69_RS14430	type IV pilus modification protein PilV	1.335100289	5.41E-185	5.09E-183
E2P69_RS12845	thiol:disulfide interchange protein DsbA/DsbL	1.333550359	5.31E-46	7.10E-45
E2P69_RS14550	peptidoglycan-binding protein	1.311016051	1.32E-53	2.05E-52
E2P69_RS11355	FAD-dependent monooxygenase	1.289076501	1.35E-16	7.14E-16
E2P69_RS21105	histidinol dehydrogenase HisD	1.288574186	2.68E-60	4.79E-59
E2P69_RS20335	histidine-type phosphatase	1.286655989	7.76E-83	2.14E-81
E2P69_RS08790	acetylglutamate kinase	1.28659532	9.04E-63	1.68E-61
E2P69_RS21135	bifunctional phosphoribosyl-AMP cyclohydrolase/phosphoribosyl-ATP diphosphatase HisIE	1.278898246	2.43E-40	2.79E-39
E2P69_RS10240	PilN domain-containing protein	1.275745127	2.60E-28	2.16E-27
E2P69_RS19740	glycosyltransferase	1.274221017	9.06E-109	3.26E-107
E2P69_RS08775	argininosuccinate lyase	1.248709383	7.82E-46	1.03E-44
E2P69_RS15705	anti-sigma factor	1.247862945	4.01E-26	3.03E-25
E2P69_RS21120	imidazole glycerol phosphate synthase subunit HisH	1.247658604	7.75E-37	7.95E-36

E2P69_RS06840	hypothetical protein	1.246706559	0.00226121	0.004414794
E2P69_RS08795	acetylornithine deacetylase	1.245678481	3.92E-53	6.06E-52
E2P69_RS19900	LacI family DNA-binding transcriptional regulator	1.245041273	1.41E-33	1.34E-32
E2P69_RS08765	glutamate 5-kinase	1.239207467	3.42E-46	4.60E-45
E2P69_RS00945	hypothetical protein	1.238222276	3.35E-40	3.82E-39
E2P69_RS01265	peptidyl-dipeptidase Dcp	1.220579461	5.36E-90	1.62E-88
E2P69_RS18290	hypothetical protein	1.218430638	2.19E-18	1.24E-17
E2P69_RS21110	histidinol-phosphate transaminase HisC	1.215461622	9.19E-50	1.32E-48
E2P69_RS14545	hypothetical protein	1.211330231	8.41E-57	1.40E-55
E2P69_RS06975	helix-turn-helix domain-containing protein	1.208815397	4.45E-07	1.28E-06
E2P69_RS21130	imidazole glycerol phosphate synthase subunit HisF	1.207118916	5.25E-53	8.08E-52
E2P69_RS21010	single-stranded-DNA-specific exonuclease RecJ	1.204702778	2.76E-28	2.29E-27
E2P69_RS07785	TIGR01244 family phosphatase	1.203052086	1.44E-14	6.90E-14
E2P69_RS14345	tRNA pseudouridine(55) synthase TruB	1.200845169	4.18E-48	5.77E-47
E2P69_RS14630	DUF4194 domain-containing protein	1.200463788	4.84E-12	2.03E-11
E2P69_RS14340	30S ribosome-binding factor RbfA	1.196257593	4.37E-35	4.38E-34
E2P69_RS06805	hypothetical protein	1.195056486	1.56E-06	4.35E-06
E2P69_RS11825	50S ribosomal protein L21 rplU	1.195051764	4.71E-178	3.98E-176
E2P69_RS19910	glycoside hydrolase family 2	1.18652199	2.13E-212	2.60E-210
E2P69_RS05320	zinc-binding dehydrogenase	1.183968483	6.58E-123	2.73E-121
E2P69_RS04285	DUF3348 domain-containing protein	1.183081163	3.28E-32	3.01E-31
E2P69_RS22085	elongation factor G	1.181241039	1.23E-200	1.38E-198
E2P69_RS18285	M13 family peptidase	1.179266987	5.61E-33	5.25E-32
E2P69_RS07700	DNA-binding protein	1.174206868	2.02E-25	1.49E-24
E2P69_RS06870	lysis protein	1.173636318	4.32E-08	1.37E-07
E2P69_RS21115	bifunctional histidinol-phosphatase/imidazoleglycerol-phosphate dehydratase HisB	1.172304544	9.41E-60	1.65E-58
E2P69_RS08780	N-acetyl-gamma-glutamyl-phosphate reductase	1.17219519	4.27E-37	4.42E-36
E2P69_RS18530	phosphate ABC transporter permease subunit PstC	1.148426491	1.28E-77	3.14E-76
E2P69_RS10975	ribonuclease HII	1.148249838	1.29E-34	1.27E-33
E2P69_RS14425	GspH/FimT family pseudopilin	1.144052267	3.45E-128	1.49E-126
E2P69_RS22110	50S ribosomal protein L7/L12 rplL	1.13912098	2.12E-123	8.87E-122
E2P69_RS00060	membrane protein insertase YidC	1.133281723	3.76E-141	1.98E-139
E2P69_RS22460	50S ribosomal protein L15 rplO	1.130089947	3.53E-89	1.05E-87
E2P69_RS06850	HK97 family phage prohead protease	1.129845742	4.50E-09	1.55E-08
E2P69_RS10965	acyl-ACP--UDP-N-acetylglucosamine O-acyltransferase	1.12971771	6.84E-44	8.57E-43
E2P69_RS08055	3-deoxy-manno-octulosonate cytidylyltransferase	1.129674247	6.63E-08	2.07E-07
E2P69_RS22130	transcription termination/antitermination protein NusG	1.126854645	1.51E-97	4.85E-96
E2P69_RS22420	50S ribosomal protein L24 rplX	1.124403743	3.17E-99	1.06E-97

E2P69_RS01310	isocitrate lyase	1.114214524	3.28E-19	1.96E-18
E2P69_RS22380	50S ribosomal protein L2 rplB	1.112562169	1.77E-68	3.71E-67
E2P69_RS11260	DUF1328 domain-containing protein	1.107386111	4.68E-79	1.17E-77
E2P69_RS08700	tetratricopeptide repeat protein	1.105003035	8.63E-14	3.98E-13
E2P69_RS10970	lipid-A-disaccharide synthase	1.101092748	3.24E-44	4.10E-43
E2P69_RS18525	phosphate ABC transporter permease PstA	1.099036056	2.67E-69	5.70E-68
E2P69_RS21800	50S ribosomal protein L28 rpmB	1.097286943	3.52E-147	2.25E-145
E2P69_RS12605	endonuclease/exonuclease/phosphatase family protein	1.090163425	3.00E-116	1.17E-114
E2P69_RS07695	inovirus-type Gp2 protein	1.088052224	9.06E-18	5.07E-17
E2P69_RS18820	DUF3011 domain-containing protein	1.072803888	5.83E-65	1.12E-63
E2P69_RS01040	P-II family nitrogen regulator	1.071225061	2.81E-60	5.00E-59
E2P69_RS05670	hypothetical protein	1.068675872	9.44E-06	2.46E-05
E2P69_RS11820	50S ribosomal protein L27 rpmA	1.06578538	1.67E-70	3.64E-69
E2P69_RS18425	UDP-N-acetylmuramoyl-L-alanine--D-glutamate ligase	1.064250262	1.35E-58	2.34E-57
E2P69_RS14330	transcription termination/antitermination protein NusA	1.064167383	2.20E-122	9.01E-121
E2P69_RS22490	50S ribosomal protein L17 rplQ	1.064121103	2.58E-82	6.97E-81
E2P69_RS22385	30S ribosomal protein S19 rpsS	1.063785595	8.66E-59	1.51E-57
E2P69_RS12640	hypothetical protein	1.061375787	3.41E-79	8.62E-78
E2P69_RS09340	FecR domain-containing protein- T6 cluster gene	1.050854702	1.44E-61	2.65E-60
E2P69_RS12395	lipoprotein	1.04960942	5.97E-49	8.45E-48
E2P69_RS22430	30S ribosomal protein S14 rpsN	1.049399629	2.76E-47	3.78E-46
E2P69_RS08585	carbohydrate ABC transporter permease	1.048738271	4.59E-45	5.98E-44
E2P69_RS00065	ribonuclease P protein component	1.045825252	3.42E-57	5.79E-56
E2P69_RS21880	type VI secretion system contractile sheath small subunit- cluster III	1.045220006	6.79E-10	2.47E-09
E2P69_RS09395	type VI secretion system baseplate subunit TssK	1.03533504	5.86E-12	2.44E-11
E2P69_RS18515	phosphate signaling complex protein PhoU	1.030276966	1.91E-80	4.98E-79
E2P69_RS15315	alpha-amylase family protein	1.024607347	3.51E-144	2.05E-142
E2P69_RS22445	50S ribosomal protein L18 rplR	1.024449157	4.05E-87	1.18E-85
E2P69_RS12520	rod shape-determining protein MreC	1.017973834	7.19E-26	5.40E-25
E2P69_RS22400	50S ribosomal protein L16 rplP	1.01730704	1.80E-56	2.96E-55
E2P69_RS14325	ribosome maturation factor RimP	1.015171832	7.08E-81	1.86E-79
E2P69_RS06855	terminase large subunit	1.012629885	1.25E-13	5.71E-13
E2P69_RS07115	tRNA (adenosine(37)-N6)-dimethylallyltransferase MiaA	1.012098411	3.28E-39	3.62E-38
E2P69_RS09375	hypothetical protein- T6 associated protein	1.00716441	0.000183291	0.000407828
E2P69_RS08900	putative acyl-CoA thioester hydrolase	1.006651333	1.41E-26	1.10E-25
E2P69_RS01395	ExeM/NucH family extracellular endonuclease	1.005567147	1.01E-50	1.48E-49
E2P69_RS06580	glycoside hydrolase family 95 protein	1.003525031	7.63E-33	7.12E-32
E2P69_RS02680	chemotaxis protein CheW	1.003475826	2.32E-09	8.17E-09

E2P69_RS08785	GNAT family N-acetyltransferase	1.003073737	6.79E-18	3.82E-17
E2P69_RS11605	ribosome maturation factor RimM	1.002867707	1.73E-81	4.59E-80
E2P69_RS05035	glycoside hydrolase family 99-like domain-containing protein	1.001261896	6.08E-116	2.35E-114
E2P69_RS06560	efflux transporter outer membrane subunit	1.000654594	5.47E-40	6.21E-39
E2P69_RS11810	30S ribosomal protein S20 rpsT	0.999049005	4.37E-98	1.44E-96
E2P69_RS13425	TPM domain-containing protein	0.996910099	3.96E-61	7.13E-60
E2P69_RS03325	thiol-disulfide oxidoreductase DCC family protein	0.996441579	6.72E-05	0.000157528
E2P69_RS16410	glycerophosphodiester phosphodiesterase family protein	0.995721	5.06E-134	2.41E-132
E2P69_RS16970	malate dehydrogenase	0.995317824	5.47E-54	8.62E-53
E2P69_RS00270	DNA topoisomerase IB	0.994081854	5.34E-114	2.03E-112
E2P69_RS04005	phage major tail tube protein	0.992594687	1.13E-05	2.91E-05
E2P69_RS11240	hypothetical protein	0.991399526	2.26E-27	1.83E-26
E2P69_RS07120	dihydropteroate synthase	0.988774301	1.00E-35	1.01E-34
E2P69_RS12525	rod shape-determining protein MreD	0.988005111	7.67E-12	3.16E-11
E2P69_RS12655	BON domain-containing protein	0.982730581	3.89E-17	2.11E-16
E2P69_RS17115	SDR family oxidoreductase	0.979377948	3.03E-21	1.95E-20
E2P69_RS20655	M1 family metallopeptidase	0.977336673	3.83E-57	6.45E-56
E2P69_RS14635	DUF3375 domain-containing protein	0.974548624	1.03E-11	4.23E-11
E2P69_RS22390	50S ribosomal protein L22 rplV	0.973042213	1.64E-44	2.11E-43
E2P69_RS07515	3-phosphoshikimate 1-carboxyvinyltransferase aroA	0.97201597	7.80E-57	1.30E-55
E2P69_RS11610	30S ribosomal protein S16 rpsP	0.969515064	5.97E-74	1.41E-72
E2P69_RS22410	30S ribosomal protein S17 rpsQ	0.969057972	3.60E-61	6.51E-60
E2P69_RS13920	DUF4870 domain-containing protein	0.968939256	9.53E-34	9.07E-33
E2P69_RS13985	DUF418 domain-containing protein	0.968297402	3.42E-18	1.94E-17
E2P69_RS06160	dihydrodipicolinate synthase family protein	0.964172046	3.46E-30	3.07E-29
E2P69_RS21460	TrbI/VirB10 family protein	0.958716367	3.61E-41	4.26E-40
E2P69_RS21885	type VI secretion system contractile sheath large subunit	0.95765865	6.51E-14	3.01E-13
E2P69_RS11650	PAS domain-containing sensor histidine kinase	0.954104174	2.91E-80	7.53E-79
E2P69_RS03460	30S ribosomal protein S21 rpsU	0.953640648	6.16E-64	1.16E-62
E2P69_RS22555	hypothetical protein	0.95060851	4.28E-98	1.42E-96
E2P69_RS06015	WecB/TagA/CpsF family glycosyltransferase	0.950318673	1.17E-32	1.08E-31
E2P69_RS16105	hypothetical protein	0.949133279	1.26E-19	7.63E-19
E2P69_RS10960	3-hydroxyacyl-ACP dehydratase FabZ	0.948873502	1.29E-25	9.60E-25
E2P69_RS18520	phosphate ABC transporter ATP-binding protein PstB	0.947710356	1.28E-67	2.61E-66
E2P69_RS04615	hypothetical protein	0.942187367	1.12E-104	3.88E-103
E2P69_RS21805	50S ribosomal protein L33	0.939732263	1.74E-50	2.52E-49
E2P69_RS06800	hypothetical protein	0.938344894	0.002004886	0.003961012
E2P69_RS08745	MFS transporter	0.937512239	2.38E-12	1.02E-11
E2P69_RS16220	lamin tail domain-containing protein	0.93466808	7.26E-83	2.02E-81
E2P69_RS04620	hypothetical protein	0.930287366	2.10E-65	4.11E-64
E2P69_RS09890	acetolactate synthase	0.929279611	9.33E-30	8.14E-29
E2P69_RS19735	glycosyltransferase family 2 protein	0.926814334	1.17E-40	1.36E-39

E2P69_RS22115	50S ribosomal protein L10	0.925068255	2.39E-44	3.06E-43
E2P69_RS22560	DUF262 domain-containing protein	0.924710855	5.94E-72	1.34E-70
E2P69_RS22415	50S ribosomal protein L14	0.923844719	2.85E-55	4.58E-54
E2P69_RS10955	UDP-3-O-(3-hydroxymyristoyl)glucosamine N-acyltransferase	0.923443704	7.63E-44	9.52E-43
E2P69_RS10770	hypothetical protein	0.922091479	0.0001791	0.000398932
E2P69_RS08800	GNAT family N-acetyltransferase	0.917288501	1.42E-17	7.88E-17
E2P69_RS00300	M56 family metallopeptidase	0.915865804	1.42E-26	1.10E-25
E2P69_RS06005	glycosyltransferase	0.912741237	2.12E-64	4.02E-63
E2P69_RS00455	hypothetical protein	0.91103006	5.89E-12	2.45E-11
E2P69_RS14140	D-glycerate dehydrogenase	0.905148139	7.17E-32	6.51E-31
E2P69_RS22120	50S ribosomal protein L1	0.904923486	3.56E-83	9.97E-82
E2P69_RS12965	thiamine-phosphate kinase	0.9042295	4.88E-17	2.64E-16
E2P69_RS08905	phytoene synthase	0.902742734	5.91E-08	1.86E-07
E2P69_RS03795	LysM peptidoglycan-binding domain-containing protein	0.900991846	2.41E-78	5.97E-77
E2P69_RS10410	TIGR00730 family Rossman fold protein	0.900760938	1.50E-32	1.38E-31
E2P69_RS18060	alkaline phosphatase family protein	0.900128369	6.35E-65	1.21E-63
E2P69_RS09400	type VI secretion system protein TssL	0.899323418	2.40E-06	6.60E-06
E2P69_RS08390	hypothetical protein	0.898811411	7.14E-13	3.15E-12
E2P69_RS07980	hypothetical protein	0.898785906	3.54E-85	1.03E-83
E2P69_RS13630	DUF2007 domain-containing protein	0.898569761	2.31E-08	7.46E-08
E2P69_RS01855	PilT/PilU family type 4a pilus ATPase	0.896067908	5.39E-38	5.78E-37
E2P69_RS15635	DUF4142 domain-containing protein	0.893017136	1.57E-61	2.86E-60
E2P69_RS06565	polyamine ABC transporter ATP-binding protein PotA	0.891230352	1.62E-25	1.20E-24
E2P69_RS05045	hypothetical protein	0.889807409	6.49E-58	1.11E-56
E2P69_RS12810	multifunctional CCA addition/repair protein	0.887858082	2.81E-28	2.32E-27
E2P69_RS17150	RNA polymerase factor sigma-54	0.886301824	9.29E-57	1.54E-55
E2P69_RS08590	sugar ABC transporter permease	0.883539511	7.70E-29	6.51E-28
E2P69_RS07810	glycosyltransferase	0.882592258	2.28E-37	2.38E-36
E2P69_RS21445	P-type conjugative transfer protein TrbL	0.881994759	8.13E-40	9.18E-39
E2P69_RS17100	UDP-3-O-(3-hydroxymyristoyl)glucosamine N-acyltransferase	0.881579667	4.11E-14	1.92E-13
E2P69_RS07570	type VI secretion system-associated FHA domain protein TagH	0.880885627	3.70E-05	8.98E-05
E2P69_RS05985	acyltransferase family protein	0.877536359	3.11E-44	3.95E-43
E2P69_RS00315	hypothetical protein	0.876844565	0.026226238	0.042261884
E2P69_RS19550	DUF2069 domain-containing protein	0.873999709	3.23E-26	2.46E-25
E2P69_RS00295	BlaI/MecI/CopY family transcriptional regulator	0.872671243	2.21E-21	1.43E-20
E2P69_RS06780	hypothetical protein	0.87264071	7.43E-05	0.000173386
E2P69_RS18440	bifunctional aspartate kinase/diaminopimelate decarboxylase	0.871417714	1.79E-97	5.69E-96
E2P69_RS10950	hypothetical protein	0.87137057	6.27E-22	4.14E-21

E2P69_RS07710	hypothetical protein	0.871042249	1.70E-11	6.85E-11
E2P69_RS16985	DUF2127 domain-containing protein	0.870353691	1.88E-15	9.52E-15
E2P69_RS04810	F0F1 ATP synthase subunit epsilon	0.869701457	1.81E-43	2.24E-42
E2P69_RS05930	50S ribosomal protein L20 rplT	0.868350733	4.55E-63	8.54E-62
E2P69_RS12935	bifunctional diaminohydroxyphosphoribosylaminopyrimidine deaminase/5-amino-6-(5-phosphoribosylamino)uracil reductase RibD	0.865458675	6.89E-25	4.99E-24
E2P69_RS19555	xylanase	0.863664045	6.03E-17	3.25E-16
E2P69_RS02695	methyl-accepting chemotaxis protein	0.862728074	7.47E-36	7.54E-35
E2P69_RS17110	NeuD/PglB/VioB family sugar acetyltransferase	0.862722194	1.84E-17	1.01E-16
E2P69_RS05925	50S ribosomal protein L35 rpmL	0.862056367	1.44E-37	1.52E-36
E2P69_RS09740	energy transducer TonB	0.862041066	4.73E-13	2.10E-12
E2P69_RS06685	DUF1275 domain-containing protein	0.860770063	9.73E-10	3.51E-09
E2P69_RS05935	phenylalanine--tRNA ligase subunit alpha pheS	0.860449764	1.96E-26	1.50E-25
E2P69_RS09990	membrane protein	0.860296775	4.61E-43	5.65E-42
E2P69_RS02690	Hpt domain-containing protein	0.858433434	2.49E-66	5.01E-65
E2P69_RS04455	hypothetical protein	0.854966509	3.26E-12	1.39E-11
E2P69_RS09280	ABC transporter permease subunit	0.854388947	1.89E-18	1.08E-17
E2P69_RS04860	efflux RND transporter periplasmic adaptor subunit	0.85386187	5.73E-09	1.96E-08
E2P69_RS22395	30S ribosomal protein S3 rpsC	0.853366049	1.36E-48	1.92E-47
E2P69_RS01165	MFS transporter	0.85299061	2.29E-23	1.59E-22
E2P69_RS09410	type VI secretion system-associated protein TagF	0.852984118	0.022791224	0.037160189
E2P69_RS22125	50S ribosomal protein L11 rplK	0.852785923	1.79E-61	3.26E-60
E2P69_RS07575	type VI secretion system baseplate subunit TssK	0.852474295	0.001385768	0.002787009
E2P69_RS06810	DUF3168 domain-containing protein	0.850428813	9.45E-07	2.66E-06
E2P69_RS22375	50S ribosomal protein L23 rplW	0.846578731	5.64E-46	7.52E-45
E2P69_RS14565	FG-GAP repeat protein	0.846078309	2.32E-28	1.94E-27
E2P69_RS21450	type IV secretory pathway, TrbF protein	0.845821844	5.72E-23	3.90E-22
E2P69_RS03935	hypothetical protein	0.844387002	0.001568701	0.003136644
E2P69_RS17085	glycosyltransferase	0.842907429	3.47E-32	3.17E-31
E2P69_RS07800	GMC family oxidoreductase	0.842306602	2.21E-20	1.38E-19
E2P69_RS00195	S41 family peptidase	0.838570252	3.34E-69	7.10E-68
E2P69_RS11645	response regulator	0.836921295	1.90E-37	2.00E-36
E2P69_RS11135	DUF1365 domain-containing protein	0.836231862	4.70E-13	2.09E-12
E2P69_RS06825	phage gp6-like head-tail connector protein	0.83535177	9.27E-06	2.42E-05
E2P69_RS09345	RNA polymerase sigma factor	0.835303355	1.22E-82	3.34E-81
E2P69_RS22440	50S ribosomal protein L6 rplF	0.833418325	2.01E-73	4.64E-72
E2P69_RS11345	ligase-associated DNA damage response DEXH box helicase	0.83078192	3.17E-22	2.12E-21
E2P69_RS13175	hypothetical protein	0.829486166	2.09E-33	1.96E-32
E2P69_RS22425	50S ribosomal protein L5 rplE	0.828731574	2.35E-55	3.79E-54
E2P69_RS07720	DUF2523 domain-containing protein	0.826335863	0.0286568	0.045804446
E2P69_RS17095	FkbM family methyltransferase	0.824124355	2.72E-27	2.19E-26
E2P69_RS21530	tRNA uridine-5-	0.823905012	3.40E-58	5.84E-57

	carboxymethylaminomethyl(34) synthesis enzyme MnmG			
E2P69_RS11350	ligase-associated DNA damage response endonuclease PdeM	0.823873089	3.05E-08	9.77E-08
E2P69_RS22135	preprotein translocase subunit SecE	0.823722346	3.14E-51	4.67E-50
E2P69_RS15795	tryptophan--tRNA ligase	0.823662847	9.95E-67	2.02E-65
E2P69_RS00440	CsbD family protein	0.822209096	2.10E-44	2.70E-43
E2P69_RS08015	hypothetical protein	0.821970586	1.16E-38	1.27E-37
E2P69_RS22850	hypothetical protein	0.821726524	0.014596314	0.024790424
E2P69_RS08595	sugar ABC transporter substrate-binding protein	0.820559169	4.34E-27	3.47E-26
E2P69_RS18535	phosphate ABC transporter substrate-binding protein PstS	0.819249842	1.81E-48	2.53E-47
E2P69_RS05990	glycosyltransferase family 4 protein	0.818689608	8.10E-59	1.42E-57
E2P69_RS05940	phenylalanine--tRNA ligase subunit beta	0.81834031	1.01E-34	1.00E-33
E2P69_RS17425	outer membrane protein assembly factor BamB	0.817922614	1.78E-57	3.03E-56
E2P69_RS09900	ketol-acid reductoisomerase ilvC	0.815772917	1.52E-73	3.54E-72
E2P69_RS11595	50S ribosomal protein L19 rplC	0.815125443	3.14E-57	5.33E-56
E2P69_RS05995	glycosyltransferase	0.813054672	1.16E-34	1.14E-33
E2P69_RS12835	endonuclease/exonuclease/phosphatase family protein	0.81294404	2.16E-34	2.10E-33
E2P69_RS06555	multidrug efflux MFS transporter	0.811401637	5.11E-50	7.35E-49
E2P69_RS22210	SDR family NAD(P)-dependent oxidoreductase	0.809337843	8.56E-20	5.22E-19
E2P69_RS17120	SDR family oxidoreductase	0.808902944	1.18E-18	6.82E-18
E2P69_RS02685	hypothetical protein	0.804212234	1.26E-11	5.12E-11
E2P69_RS13865	DUF839 domain-containing protein	0.802953312	2.96E-37	3.09E-36
E2P69_RS09230	tRNA (N6-isopentenyl adenosine(37)-C2)-methylthiotransferase MiaB	0.802714582	4.00E-44	5.05E-43
E2P69_RS19460	aminoacyl-tRNA hydrolase pth	0.802662643	1.28E-23	8.96E-23
E2P69_RS21910	type VI secretion system baseplate subunit TssF	0.80064144	6.32E-08	1.98E-07
E2P69_RS22825	mannose-1-phosphate guanylyltransferase/mannose-6-phosphate isomerase	0.799893656	5.11E-68	1.06E-66
E2P69_RS10505	type II toxin-antitoxin system RatA family toxin	0.799731264	5.51E-30	4.87E-29
E2P69_RS06010	polysaccharide pyruvyl transferase family protein	0.799513515	6.46E-43	7.89E-42
E2P69_RS11925	DNA-3-methyladenine glycosylase 2 family protein	0.799449996	1.81E-12	7.78E-12
E2P69_RS10245	type 4a pilus biogenesis protein PilO	0.799244708	1.87E-17	1.03E-16
E2P69_RS18510	RcnB family protein	0.798857771	1.49E-26	1.15E-25
E2P69_RS14625	ATP-binding protein	0.797894835	1.27E-11	5.15E-11
E2P69_RS21390	hypothetical protein	0.796596095	5.27E-33	4.94E-32
E2P69_RS06000	lipopolysaccharide biosynthesis protein	0.796288851	4.94E-39	5.43E-38
E2P69_RS01305	malate synthase A	0.794222299	7.21E-11	2.78E-10
E2P69_RS19505	tetratricopeptide repeat protein	0.793885446	1.93E-23	1.34E-22
E2P69_RS21435	conjugal transfer protein	0.791811231	5.99E-36	6.07E-35

E2P69_RS13385	FtsX-like permease family protein	0.790864247	1.83E-26	1.42E-25
E2P69_RS17430	ribosome biogenesis GTPase Der	0.790452712	9.53E-42	1.14E-40
E2P69_RS10235	pilus assembly protein PilM	0.790037006	1.25E-27	1.02E-26
E2P69_RS17105	Rieske 2Fe-2S domain-containing protein	0.788917316	1.06E-14	5.17E-14
E2P69_RS11930	DUF423 domain-containing protein	0.787614026	4.58E-06	1.23E-05
E2P69_RS05030	ABC transporter ATP-binding protein	0.786963372	7.10E-47	9.67E-46
E2P69_RS08995	alpha/beta hydrolase	0.784565309	1.90E-21	1.23E-20
E2P69_RS18435	hypothetical protein	0.784447516	3.91E-26	2.96E-25
E2P69_RS05680	RHS repeat-associated core domain-containing protein	0.7838263	1.56E-20	9.81E-20
E2P69_RS16415	TonB-dependent receptor	0.78188223	6.47E-82	1.73E-80
E2P69_RS11800	bifunctional riboflavin kinase/FAD synthetase	0.780832788	5.81E-38	6.22E-37
E2P69_RS09895	acetolactate synthase 2 catalytic subunit	0.780781297	5.89E-37	6.05E-36
E2P69_RS21920	AAA family ATPase	0.780544636	0.000162729	0.000365017
E2P69_RS05785	L-fuconate dehydratase	0.777806625	1.98E-07	5.93E-07
E2P69_RS21630	glycerol kinase GlpK	0.774086598	7.45E-26	5.59E-25
E2P69_RS14145	aspartate-semialdehyde dehydrogenase	0.773608801	3.74E-15	1.87E-14
E2P69_RS21340	DNA-binding protein	0.772954966	1.34E-09	4.79E-09
E2P69_RS05040	hypothetical protein	0.771231383	7.73E-31	6.95E-30
E2P69_RS11145	DUF2878 domain-containing protein	0.769713289	1.60E-05	4.05E-05
E2P69_RS21425	VirB3 family type IV secretion system protein	0.766799114	3.15E-30	2.79E-29
E2P69_RS05050	GtrA family protein	0.766533548	4.71E-16	2.45E-15
E2P69_RS07805	alpha/beta hydrolase	0.76576649	5.35E-27	4.26E-26
E2P69_RS11835	acyl-CoA thioesterase	0.764576564	1.32E-21	8.65E-21
E2P69_RS21430	DUF853 family protein	0.764362118	9.01E-39	9.87E-38
E2P69_RS01215	DUF1328 domain-containing protein	0.764253514	1.91E-26	1.47E-25
E2P69_RS05520	xylose isomerase xylA	0.763040697	7.53E-17	4.04E-16
E2P69_RS17125	ketoacyl-ACP synthase III	0.763023555	3.13E-23	2.16E-22
E2P69_RS04520	UdgX family uracil-DNA binding protein	0.760039667	1.82E-11	7.28E-11
E2P69_RS06860	P27 family phage terminase small subunit	0.759944348	0.000822736	0.001693291
E2P69_RS21350	hypothetical protein	0.758793209	9.50E-40	1.07E-38
E2P69_RS04675	alpha/beta fold hydrolase	0.757034796	0.000425374	0.000904448
E2P69_RS07545	hypothetical protein	0.755634649	0.025268099	0.040940842
E2P69_RS22450	30S ribosomal protein S5 rpsE	0.755076944	2.85E-56	4.67E-55
E2P69_RS05980	acyltransferase family protein	0.754156307	8.81E-46	1.16E-44
E2P69_RS17140	sigma-54-dependent Fis family transcriptional regulator	0.751609849	9.31E-63	1.72E-61
E2P69_RS07195	hypothetical protein	0.75120439	9.18E-10	3.32E-09
E2P69_RS21025	carbamoyl-phosphate synthase large subunit CarB	0.749940507	2.03E-53	3.16E-52
E2P69_RS22370	50S ribosomal protein L4 rplD	0.749826576	9.93E-38	1.06E-36
E2P69_RS08470	glutamine-hydrolyzing GMP synthase GuaA	0.749568658	4.44E-43	5.46E-42
E2P69_RS21410	helix-turn-helix domain-containing protein	0.748741045	7.20E-30	6.31E-29

E2P69_RS12420	ATP-dependent protease ATPase subunit HslU	0.745875923	1.03E-32	9.54E-32
E2P69_RS13990	FAD-dependent oxidoreductase	0.745835004	3.23E-19	1.93E-18
E2P69_RS18885	restriction endonuclease	0.745208968	2.94E-43	3.62E-42
E2P69_RS22030	GGDEF domain-containing protein	0.744465354	3.41E-36	3.46E-35
E2P69_RS05290	TonB-dependent receptor	0.744101531	1.08E-10	4.12E-10
E2P69_RS21735	GFA family protein	0.740420221	1.98E-26	1.52E-25
E2P69_RS16335	4-alpha-glucanotransferase	0.737503668	4.89E-27	3.90E-26
E2P69_RS22790	hypothetical protein	0.736440592	3.12E-26	2.37E-25
E2P69_RS09415	serine/threonine-protein phosphatase	0.735707956	0.015719248	0.02654906
E2P69_RS14465	excinuclease ABC subunit UvrB	0.734159198	3.69E-52	5.59E-51
E2P69_RS06760	tail assembly protein	0.732925942	0.00984622	0.017321822
E2P69_RS04105	GPO family capsid scaffolding protein	0.730541439	0.005760218	0.010597061
E2P69_RS21900	type VI secretion protein, ImpE/SciE family	0.729598294	0.00180657	0.003594918
E2P69_RS05650	hypothetical protein	0.729266412	8.49E-13	3.73E-12
E2P69_RS19925	3-deoxy-7-phosphoheptulonate synthase	0.728595111	7.13E-27	5.63E-26
E2P69_RS16345	1,4-alpha-glucan branching enzyme	0.728575895	3.05E-74	7.26E-73
E2P69_RS09365	tetratricopeptide repeat protein	0.727515587	6.47E-05	0.000152264
E2P69_RS14115	transglycosylase SLT domain-containing protein	0.727282842	5.18E-21	3.32E-20
E2P69_RS08910	phosphoglycolate phosphatase	0.725905088	4.30E-08	1.36E-07
E2P69_RS12330	response regulator transcription factor	0.72309009	6.84E-20	4.20E-19
E2P69_RS14605	lytic murein transglycosylase	0.721751555	6.62E-29	5.61E-28
E2P69_RS22605	ImpE protein	0.720337824	0.000655052	0.00136034
E2P69_RS18235	transcription-repair coupling factor mfd	0.718394689	8.01E-72	1.79E-70
E2P69_RS07430	cytochrome c-type biogenesis protein CcmH	0.717614018	0.007431365	0.013391973
E2P69_RS04325	ABC transporter ATP-binding protein	0.717250004	1.04E-13	4.76E-13
E2P69_RS11745	DUF805 domain-containing protein	0.715612502	1.31E-34	1.28E-33
E2P69_RS07790	sulfite exporter TauE/SafE family protein	0.71382078	1.06E-11	4.36E-11
E2P69_RS15765	EamA family transporter RarD	0.712595422	3.18E-13	1.42E-12
E2P69_RS08890	pyridoxamine 5'-phosphate oxidase family protein	0.711934344	5.99E-51	8.83E-50
E2P69_RS07420	heme lyase CcmF/NrfE family subunit	0.709222945	7.76E-09	2.61E-08
E2P69_RS15480	hypothetical protein	0.707537965	1.52E-06	4.24E-06
E2P69_RS17475	glutathione-dependent formaldehyde dehydrogenase	0.706672747	1.66E-15	8.42E-15
E2P69_RS21330	carbon storage regulator CsrA	0.70629351	4.08E-25	2.97E-24
E2P69_RS14335	translation initiation factor IF-2 infB	0.702391223	2.48E-69	5.32E-68
E2P69_RS17350	DNA translocase FtsK	0.70216324	5.75E-46	7.63E-45
E2P69_RS21030	hypothetical protein	0.70196624	2.70E-09	9.47E-09
E2P69_RS15860	type III pantothenate kinase	0.701042142	8.04E-06	2.11E-05
E2P69_RS16340	malto-oligosyltrehalose trehalohydrolase TreZ	0.699348097	1.48E-36	1.51E-35
E2P69_RS08575	endonuclease/exonuclease/phosphata	0.697170664	2.41E-10	8.99E-10

	se family protein			
E2P69_RS09370	type VI secretion system tip protein VgrG TssI	0.696688932	6.78E-15	3.34E-14
E2P69_RS22810	hypothetical protein	0.6953028	7.36E-06	1.94E-05
E2P69_RS04445	histidine kinase	0.692523565	1.68E-32	1.55E-31
E2P69_RS21905	type VI secretion system baseplate subunit TssE	0.691607789	0.021344038	0.034910521
E2P69_RS11575	RNA-binding S4 domain-containing protein	0.691030497	3.86E-14	1.81E-13
E2P69_RS11130	FAD-dependent oxidoreductase	0.689168518	2.25E-15	1.13E-14
E2P69_RS22405	50S ribosomal protein L29 rpmC	0.688394987	1.39E-14	6.69E-14
E2P69_RS09750	cupin-like domain-containing protein	0.68584445	0.00016808	0.000376205
E2P69_RS19890	M20/M25/M40 family metallo-hydrolase	0.685778719	2.40E-38	2.60E-37
E2P69_RS04190	hypothetical protein	0.683628912	2.44E-06	6.71E-06
E2P69_RS10225	hypothetical protein	0.682987459	7.14E-35	7.11E-34
E2P69_RS15980	response regulator	0.682549949	9.33E-27	7.34E-26
E2P69_RS15790	MBL fold metallo-hydrolase	0.681680077	2.08E-38	2.26E-37
E2P69_RS14695	cardiolipin synthase B	0.679684016	2.02E-26	1.54E-25
E2P69_RS20330	siderophore-interacting protein	0.678982254	9.95E-10	3.58E-09
E2P69_RS19115	transposase	0.678438586	0.000111454	0.000255253
E2P69_RS16895	sulfate/molybdate ABC transporter ATP-binding protein	0.678271811	4.05E-26	3.05E-25
E2P69_RS17695	type VI secretion system contractile sheath large subunit TssC	0.677566519	0.015181478	0.025693163
E2P69_RS14540	DUF4189 domain-containing protein	0.676903503	6.98E-24	4.91E-23
E2P69_RS09360	PAAR domain-containing protein	0.675971221	0.018285147	0.030399423
E2P69_RS08255	FAD-binding oxidoreductase	0.674944769	9.75E-20	5.94E-19
E2P69_RS08695	cytochrome c-type biogenesis protein CcmH	0.674421308	0.00213978	0.004201473
E2P69_RS06770	phage minor tail protein L	0.674383466	9.58E-06	2.49E-05
E2P69_RS21020	transcription elongation factor GreA	0.67260166	1.29E-11	5.22E-11
E2P69_RS21740	GlsB/YeaQ/YmgE family stress response membrane protein	0.671438163	1.30E-24	9.37E-24
E2P69_RS15320	TonB-dependent receptor	0.6712537	5.23E-74	1.24E-72
E2P69_RS16795	phosphate regulon sensor histidine kinase PhoR	0.670257879	3.62E-22	2.41E-21
E2P69_RS13940	tRNA lysidine(34) synthetase TilS	0.669084322	3.58E-09	1.24E-08
E2P69_RS14155	VOC family protein	0.668811065	9.38E-06	2.44E-05
E2P69_RS15510	arginine decarboxylase speA	0.667339791	3.74E-55	5.98E-54
E2P69_RS21890	type VI secretion system tube protein Hcp	0.667289552	6.61E-07	1.88E-06
E2P69_RS16690	peptidyl-prolyl cis-trans isomerase	0.665658988	7.77E-43	9.45E-42
E2P69_RS08855	VOC family protein	0.664297405	4.84E-12	2.03E-11
E2P69_RS09260	DUF4105 domain-containing protein	0.663665237	7.69E-24	5.39E-23
E2P69_RS09440	type VI secretion system protein TssA	0.663169212	3.49E-05	8.50E-05
E2P69_RS07520	prephenate dehydratase pheA	0.662733043	2.48E-33	2.33E-32
E2P69_RS21535	DUF2490 domain-containing protein	0.661840017	5.56E-14	2.59E-13
E2P69_RS17340	N-acetyltransferase	0.660663479	4.47E-12	1.88E-11
E2P69_RS05860	DUF485 domain-containing protein	0.658768666	1.63E-19	9.86E-19
E2P69_RS21420	TrbC/VirB2 family protein	0.658436245	4.25E-22	2.82E-21
E2P69_RS08580	discoidin domain-containing protein	0.658434875	1.42E-30	1.27E-29

E2P69_RS06235	S9 family peptidase	0.657078327	7.61E-11	2.93E-10
E2P69_RS05025	ABC transporter permease	0.655547042	9.76E-33	9.06E-32
E2P69_RS18835	DNA gyrase subunit A gyrA	0.654593085	1.26E-55	2.03E-54
E2P69_RS22365	50S ribosomal protein L3 rplC	0.653599285	6.84E-27	5.41E-26
E2P69_RS17420	tetratricopeptide repeat protein	0.653405446	6.11E-41	7.17E-40
E2P69_RS03970	N-acetylmuramoyl-L-alanine amidase	0.651734057	1.65E-29	1.43E-28
E2P69_RS08635	pseudouridine synthase	0.650922928	3.56E-15	1.78E-14
E2P69_RS07220	S10 family peptidase	0.650547307	1.10E-41	1.31E-40
E2P69_RS20770	HAD family hydrolase	0.649554963	1.11E-30	9.93E-30
E2P69_RS15720	cytochrome b	0.648234106	1.45E-17	8.02E-17
E2P69_RS06675	PhoH family protein	0.646329318	1.20E-11	4.89E-11
E2P69_RS04185	hypothetical protein	0.643160647	8.48E-07	2.40E-06
E2P69_RS11795	isoleucine--tRNA ligase ileS	0.642825978	1.42E-33	1.34E-32
E2P69_RS03445	YihY/virulence factor BrkB family protein	0.642705594	3.68E-38	3.97E-37
E2P69_RS11700	histidine kinase	0.642007118	1.09E-15	5.58E-15
E2P69_RS00055	polysaccharide deacetylase family protein	0.641944131	2.14E-08	6.93E-08
E2P69_RS06835	phage portal protein	0.640719442	1.88E-07	5.66E-07
E2P69_RS15485	SDR family NAD(P)-dependent oxidoreductase	0.640575127	6.47E-11	2.51E-10
E2P69_RS13655	hypothetical protein	0.640126979	9.59E-09	3.21E-08
E2P69_RS05865	cation acetate symporter	0.639137345	1.16E-24	8.37E-24
E2P69_RS15690	AI-2E family transporter	0.63335789	2.28E-25	1.68E-24
E2P69_RS05645	hypothetical protein	0.632427342	3.98E-14	1.86E-13
E2P69_RS21415	Flp pilus assembly complex ATPase component TadA	0.632251206	1.09E-33	1.03E-32
E2P69_RS06090	hypothetical protein	0.629509492	2.86E-19	1.71E-18
E2P69_RS16500	aminodeoxychorismate synthase component I	0.629313994	1.36E-08	4.51E-08
E2P69_RS04855	ABC transporter ATP-binding protein	0.628899205	8.13E-08	2.52E-07
E2P69_RS08555	agmatine deiminase family protein	0.628703127	3.96E-24	2.81E-23
E2P69_RS11485	acyl-CoA dehydrogenase family protein	0.627780557	2.47E-15	1.24E-14
E2P69_RS08760	glutamate-5-semialdehyde dehydrogenase	0.627765414	1.12E-14	5.41E-14
E2P69_RS00070	50S ribosomal protein L34 rpmH	0.626493213	0.028114505	0.044989723
E2P69_RS07105	GTPase HflX	0.625828598	2.93E-21	1.89E-20
E2P69_RS07830	class I SAM-dependent methyltransferase	0.624416607	0.007280744	0.013154872
E2P69_RS03820	O-antigen ligase family protein	0.62425263	9.00E-13	3.94E-12
E2P69_RS11235	Hsp70 family protein	0.623869014	6.65E-11	2.57E-10
E2P69_RS11635	galactose mutarotase	0.623571654	3.54E-21	2.28E-20
E2P69_RS10250	pilus assembly protein PilP	0.623360463	1.84E-08	6.02E-08
E2P69_RS21245	hypothetical protein	0.623052159	2.57E-10	9.56E-10
E2P69_RS21200	hypothetical protein	0.622771555	3.97E-09	1.37E-08
E2P69_RS00045	phosphatase PAP2 family protein	0.622654549	1.96E-13	8.87E-13
E2P69_RS02020	nucleotidyltransferase family protein	0.622491456	8.08E-08	2.51E-07
E2P69_RS01585	oxidoreductase	0.620071351	6.69E-19	3.90E-18
E2P69_RS04705	ABC transporter permease	0.619646841	3.50E-06	9.45E-06
E2P69_RS07715	A coat protein	0.61951836	0.006800155	0.012345768

E2P69_RS18315	hypothetical protein	0.619288915	1.43E-20	9.02E-20
E2P69_RS13640	MAPEG family protein	0.618587523	7.59E-16	3.89E-15
E2P69_RS21185	FAD-binding oxidoreductase	0.618480481	2.40E-27	1.93E-26
E2P69_RS01175	hypothetical protein	0.618229401	1.77E-19	1.06E-18
E2P69_RS04850	ABC transporter permease	0.617961763	6.43E-10	2.35E-09
E2P69_RS08540	50S ribosomal protein L36 rpmJ	0.617673124	1.14E-21	7.48E-21
E2P69_RS13255	phosphoenolpyruvate carboxylase ppc	0.615863789	5.98E-24	4.22E-23
E2P69_RS16180	hypothetical protein	0.615142672	8.83E-05	0.000204528
E2P69_RS16130	M4 family metallopeptidase	0.614588007	3.69E-25	2.69E-24
E2P69_RS11105	hypothetical protein	0.61413362	0.000283677	0.000618566
E2P69_RS22455	50S ribosomal protein L30 rpmD	0.613905536	2.63E-20	1.64E-19
E2P69_RS12645	decarboxylating 6-phosphogluconate dehydrogenase gnd	0.612687064	1.91E-29	1.64E-28
E2P69_RS17130	acyl carrier protein	0.61241244	6.44E-11	2.50E-10
E2P69_RS13420	LemA family protein	0.611602715	1.28E-30	1.15E-29
E2P69_RS07585	type VI secretion system membrane subunit TssM	0.611244739	4.46E-05	0.000107039
E2P69_RS12505	sigma-54-dependent Fis family transcriptional regulator	0.610798243	2.19E-11	8.72E-11
E2P69_RS07625	two-component system VirA-like sensor kinase	0.610796838	1.94E-22	1.31E-21
E2P69_RS08385	Cache 3/Cache 2 fusion domain-containing protein	0.610372301	6.30E-22	4.15E-21
E2P69_RS10905	elongation factor Ts	0.608578934	3.76E-37	3.90E-36
E2P69_RS18840	membrane-bound PQQ-dependent dehydrogenase, glucose/quinate/shikimate family	0.60797336	3.47E-37	3.61E-36
E2P69_RS12535	rod shape-determining protein RodA	0.606653111	3.07E-12	1.31E-11
E2P69_RS00330	TonB-dependent receptor	0.606261105	9.52E-46	1.25E-44
E2P69_RS05295	TonB-dependent receptor	0.605315521	2.31E-26	1.77E-25
E2P69_RS21300	hypothetical protein	0.604838061	2.80E-09	9.81E-09
E2P69_RS17890	glycoside hydrolase family 15 protein	0.60466798	4.57E-19	2.70E-18
E2P69_RS15325	MFS transporter	0.603715608	5.95E-35	5.94E-34
E2P69_RS15715	catalase family peroxidase	0.603169422	9.51E-23	6.44E-22
E2P69_RS16330	malto-oligosyltrehalose synthase treY	0.603148295	1.09E-25	8.16E-25
E2P69_RS05975	hypothetical protein	0.601624876	3.84E-41	4.52E-40
E2P69_RS04800	F0F1 ATP synthase subunit gamma atpG	0.601558557	1.14E-28	9.60E-28
E2P69_RS06505	MHS family MFS transporter	0.600271996	5.47E-12	2.28E-11
E2P69_RS14825	hypothetical protein	0.598532862	5.34E-34	5.12E-33
E2P69_RS08060	tetraacyldisaccharide 4'-kinase LpxK	0.598126994	5.81E-05	0.000137312
E2P69_RS09265	magnesium and cobalt transport protein CorA	0.598006219	1.23E-17	6.82E-17
E2P69_RS10705	ABC transporter ATP-binding protein	0.597684972	6.32E-21	4.02E-20
E2P69_RS21345	AAA family ATPase	0.595323859	1.91E-26	1.47E-25
E2P69_RS11685	TonB-dependent receptor	0.595257554	6.05E-27	4.80E-26
E2P69_RS15270	hypothetical protein	0.594634385	2.14E-07	6.40E-07
E2P69_RS18785	50S ribosomal protein L9 rplL	0.593248483	9.83E-18	5.48E-17
E2P69_RS09420	protein kinase	0.592840649	1.20E-05	3.09E-05

E2P69_RS10305	M23 family metallopeptidase	0.592653248	1.65E-13	7.50E-13
E2P69_RS16280	efflux RND transporter periplasmic adaptor subunit	0.592255321	0.000943018	0.001930298
E2P69_RS12115	hypothetical protein	0.589808834	2.27E-06	6.25E-06
E2P69_RS00975	phospholipase D family protein	0.58883483	1.92E-28	1.61E-27
E2P69_RS05220	bifunctional DNA-formamidopyrimidine glycosylase/DNA-(apurinic or apyrimidinic site) lyase mutM	0.587743029	3.70E-16	1.93E-15
E2P69_RS15545	P-II family nitrogen regulator	0.587104322	2.14E-07	6.40E-07
E2P69_RS12530	penicillin-binding protein 2 mrdA	0.585323263	5.85E-17	3.16E-16
E2P69_RS02285	MFS transporter	0.585254867	1.45E-06	4.05E-06
E2P69_RS07705	hypothetical protein	0.583979302	7.00E-09	2.36E-08
E2P69_RS11830	excinuclease ABC subunit UvrA	0.582796099	2.68E-29	2.29E-28
E2P69_RS04590	hypothetical protein	0.582210667	1.20E-18	6.92E-18
E2P69_RS17750	oligopeptide transporter, OPT family	0.581253271	3.86E-16	2.01E-15
E2P69_RS16085	GNAT family N-acetyltransferase	0.580237268	6.01E-19	3.52E-18

Table S2 List of genes downregulated at 8h but upregulated at 16h in the *tssM* mutant strain.

Locus tag	Gene	Product	Log2foldchange	
			8h	16h
E2P69_RS10240		PilN domain-containing protein	-0.90	1.27
E2P69_RS00945		hypothetical protein	-0.66	1.24
E2P69_RS02680	*cheW*	chemotaxis protein	-0.84	1.00
E2P69_RS17115		SDR family oxidoreductase	-0.73	0.98
E2P69_RS13920		DUF4870 domain-containing protein	-0.63	0.97
E2P69_RS08390		hypothetical protein	-1.26	0.90
E2P69_RS01855		PilT/PilU family type 4a pilus ATPase	-0.69	0.89
E2P69_RS17100		UDP-3-O-(3-hydroxymyristoyl)glucosamine N-acyltransferase	-0.62	0.88
E2P69_RS02695		methyl-accepting chemotaxis protein	-0.86	0.86
E2P69_RS17110		NeuD/PglB/VioB family sugar acetyltransferase	-0.60	0.86
E2P69_RS02690		Hpt domain-containing protein	-1.04	0.86
E2P69_RS17095		FkbM family methyltransferase	-0.70	0.82
E2P69_RS17120		SDR family oxidoreductase	-0.75	0.81
E2P69_RS02685		hypothetical protein	-0.95	0.80
E2P69_RS10245	*pilO*	type 4a pilus biogenesis protein	-0.83	0.80
E2P69_RS10235	*pilM*	pilus assembly protein	-0.80	0.79
E2P69_RS17105		Rieske 2Fe-2S domain-containing protein	-0.68	0.79
E2P69_RS17125		ketoacyl-ACP synthase III	-0.66	0.76
E2P69_RS22030		GGDEF domain-containing protein	-1.81	0.74
E2P69_RS10225		hypothetical protein	-0.65	068
E2P69_RS10250	*pilP*	pilus assembly protein	-1.02	0.62
E2P69_RS08385		Cache 3/Cache 2 fusion domain-containing protein	-1.41	0.61
E2P69_RS00330		TonB-dependent receptor	-0.61	0.60

Table S3 List of genes differentially expressed in the mutant strain associated with motility and chemotaxis

Locus tag	Gene	Product	Log2foldchange	
			8h	16h
E2P69_RS17180	*flgL*	Flagellin	-3.99	-1.75
E2P69_RS17255	*flgM*	Flagellar biosynthesis anti-sigma factor	-3.96	-2.47
E2P69_RS17260	*flgN*	Flagella protein	-3.78	-2.16
E2P69_RS17170	*fliS*	Flagellar export chaperone	-3.67	-1.48
E2P69_RS22535	*motD*	Flagellar motor protein	-3.60	-1.89
E2P69_RS22540	-	Flagellar motor protein	-3.40	-1.69
E2P69_RS04575	*motA*	Flagellar motor stator protein	-3.14	-0.74
E2P69_RS04570	*motB*	Flagellar motor protein	-3.06	-0.11*
E2P69_RS20890	-	Flagellar brake protein	-2.84	-0.89
E2P69_RS22060	*fliA*	RNA polymerase sigma factor	-2.61	-0.79
E2P69_RS17175	*fliD*	Flagellar filament capping protein	-2.56	-1.50
E2P69_RS17185	*flgL*	Flagellar hook-associated protein	-1.90	-0.52*
E2P69_RS17190	*flgK*	Flagellar hook-associated protein	-1.61	-0.80
E2P69_RS17195	*flgJ*	Flagellar assembly peptidoglycan hydrolase	-1.45	-0.88
E2P69_RS17225	*flgE*	Flagellar hook protein	-1.39	-0.36*
E2P69_RS17220	*flgF*	Flagellar basal-body rod protein	-1.26	-0.26*
E2P69_RS17200	*flgI*	Flagellar basal body P-ring protein	-1.24	-0.45*
E2P69_RS17230	*flgD*	Flagellar basal body rod modification protein	-1.13	-1.08
E2P69_RS22050	*flhF*	flagellar biosynthesis protein	-1.11	-1.79
E2P69_RS22045	*flhA*	Flagellar biosynthesis protein	-1.08	-1.32
E2P69_RS17205	*flgH*	Flagellar basal body L-ring protein	-1.04	-0.84
E2P69_RS17035	*fliN*	Flagellar motor switch protein	-1.00	-0.35*
E2P69_RS17235	*flgC*	Flagellar basal body rod protein	-1.00	-1.82
E2P69_RS17250	*flgA*	Flagellar basal body P-ring formation protein	-0.99	-1.93
E2P69_RS17055	*fliJ*	Flagellar export protein	-0.93	-0.49*
E2P69_RS17030	*fliO*	Flagellar biosynthetic protein	-0.86	-2.04
E2P69_RS17065	*fliH*	Flagellar assembly protein	-0.86	-0.34*
E2P69_RS17025	*fliP*	Flagellar type III secretion system pore protein	-0.81	-0.51
E2P69_RS17050	*fliK*	Flagellar hook-length control protein	-0.79	-0.31
E2P69_RS17070	*fliG*	flagellar motor switch protein	-0.76	-0.43*
E2P69_RS17075	*fliF*	Flagellar M-ring protein	-0.73	-1.31
E2P69_RS17080	*fliE*	flagellar hook-basal body complex protein	-0.58	-1.90
E2P69_RS17240	*flgB*	flagellar basal body rod protein	-0.52*	-2.16
E2P69_RS22040	*flhB*	flagellar biosynthesis protein	-0.54*	-2.09
E2P69_RS22010	*fliQ*	flagellar biosynthetic protein	-0.24*	-2.08
E2P69_RS17210	*flgG*	flagellar basal-body rod protein	-0.54*	-1.90
E2P69_RS17045	*fliL*	flagellar basal body associated FliL family protein	-0.31*	-1.71
E2P69_RS22015	*fliR*	flagellar biosynthetic protein	-0.40*	-1.13

Table S4 List of genes differentially expressed in the mutant strain associated with pili biosynthesis.

Locus tag	Gene	Product	Log2foldchange	
			8h	16h
E2P69_RS10895		molecular chaperone	-3.03	-0.25*
E2P69_RS17160		PilZ domain-containing protein	-2.77	-0.87
E2P69_RS19765	*pilA*	pilin PilA	-1.91	0.25*
E2P69_RS10240		PilN domain-containing protein	-0.90	1.27
E2P69_RS10250	*pilP*	pilus assembly protein PilP	-1.02	0.62
E2P69_RS10255		type IV pilus secretin PilQ family protein	-0.83	0.40*
E2P69_RS10245	*pilO*	type 4a pilus biogenesis protein PilO	-0.83	0.79
E2P69_RS10235	*pilM*	pilus assembly protein PilM	-0.80	0.79
E2P69_RS02710	*pilG*	twitching motility response regulator PilG	-0.75	0.04*
E2P69_RS01855		PilT/PilU family type 4a pilus ATPase	-0.68	0.89
E2P69_RS01860	*pilT*	type IV pilus twitching motility protein	-0.66	0.45*
E2P69_RS19730	*pilB*	type IV-A pilus assembly ATPase PilB	-0.62	-0.23*
E2P69_RS04995		PilT/PilU family type 4a pilus ATPase	-0.60	0.37*
E2P69_RS14450	*pilE*	type IV pilin protein PilE	1.45	-0.08*
E2P69_RS14445		pilus assembly protein	2.55	1.41
E2P69_RS14440	*pilX*	Tfp pilus assembly protein PilX	2.77	1.64
E2P69_RS14435		PilW family protein	2.80	1.46
E2P69_RS14430	*pilV*	type IV pilus modification protein PilV	2.96	1.33
E2P69_RS10150		PilZ domain-containing protein	-0.22*	-0.72
E2P69_RS21415	*tadA*	Flp pilus assembly complex ATPase component TadA	0.31*	0.63
E2P69_RS19760		pilin	-0.20*	1.61

* Not significant

E2P69_RS17040	*fliM*	flagellar motor switch protein	-0.49*	-0.96
E2P69_RS18255		methyl-accepting chemotaxis protein	-4.85	-1.74
E2P69_RS18260	*cheW*	purine-binding chemotaxis protein	-4.75	-1.66
E2P69_RS20895	*cheW*	chemotaxis protein	-4.47	-1.93
E2P69_RS18250	*cheA*	chemotaxis protein	-4.37	-1.48
E2P69_RS20845		MCP four helix bundle domain-containing protein	-4.32	-1.14
E2P69_RS20910	*cheR*	chemotaxis protein	-4.23	-1.70
E2P69_RS09150	*cheW*	purine-binding chemotaxis protein	-4.15	-0.83
E2P69_RS09155		MCP four helix bundle domain-containing protein	-4.08	-1.19
E2P69_RS20905		MCP four helix bundle domain-containing protein	-4.02	-1.03
E2P69_RS17295		chemotaxis protein	-4.00	-1.84
E2P69_RS22515	*cheY*	response regulator	-4.00	-1.29
E2P69_RS22510	*cheA*	chemotaxis protein	-3.95	-0.97
E2P69_RS12285		cache domain-containing protein	-3.53	-0.02*
E2P69_RS18275		chemotaxis response regulator protein-glutamate methylesterase	-3.43	-0.37*
E2P69_RS03320		methyl-accepting chemotaxis protein	-3.36	-0.61
E2P69_RS20920		chemotaxis response regulator protein-glutamate methylesterase	-3.33	-0.04*
E2P69_RS22525	*cheW*	chemotaxis protein	-3.17	-0.93
E2P69_RS20840		MCP four helix bundle domain-containing protein	-3.14	-0.54*
E2P69_RS17245	*cheV*	chemotaxis protein	-2.78	-0.77
E2P69_RS22065	*cheY*	chemotaxis response regulator	-2.65	-0.75
E2P69_RS06725		PAS domain-containing methyl-accepting chemotaxis protein	-2.64	-0.52*
E2P69_RS20915	*cheD*	chemoreceptor glutamine deamidase	-2.63	0.04*
E2P69_RS22070	*cheZ*	protein phosphatase	-2.61	-0.69
E2P69_RS22695	*cheW*	chemotaxis protein	-2.22	-0.65
E2P69_RS20825		methyl-accepting chemotaxis protein	-2.19	-0.11*
E2P69_RS20875		MCP four helix bundle domain-containing protein	-2.05	-0.19*
E2P69_RS20870		MCP four helix bundle domain-containing protein	-1.88	-0.41*
E2P69_RS09910		methyl-accepting chemotaxis protein	-1.68	-0.37*
E2P69_RS02700	*cheW*	chemotaxis protein	-1.03	-0.52*
E2P69_RS02695		methyl-accepting chemotaxis protein	-0.86	0.86
E2P69_RS02680	*cheW*	chemotaxis protein	-0.83	1.00#
E2P69_RS11665	*cheB*	chemotaxis protein	-0.74	0.11*
E2P69_RS11660	*cheR*	chemotaxis protein	-0.73	0.07*
E2P69_RS20860		MCP four helix bundle domain-containing protein	-0.70	-0.27*

* Not significant

Table S5 List of genes differentially expressed in the mutant strain associated with c-di-GMP metabolism.

Locus tag	Gene	Product	Log2foldchange	
			8h	16h
E2P69_RS18265		EAL domain-containing response regulator	-4.61	-1.46
E2P69_RS17280		EAL domain-containing protein	-4.05	-1.49
E2P69_RS06515		diguanylate cyclase	-3.51	-0.97
E2P69_RS12300		GGDEF domain-containing protein	-3.07	-0.88
E2P69_RS16355		GGDEF domain-containing protein	-3.03	-0.62
E2P69_RS13670		EAL domain-containing protein	-2.99	0.16[*]
E2P69_RS19000		EAL domain-containing protein	-2.63	-0.37[*]
E2P69_RS21675		HD-GYP domain-containing protein	-2.27	-0.53[*]
E2P69_RS22035		bifunctional diguanylate cyclase/phosphodiesterase	-2.15	0.06[*]
E2P69_RS12180		GGDEF domain-containing protein	-1.85	-0.15[*]
E2P69_RS22030		GGDEF domain-containing protein	-1.80	0.74[#]
E2P69_RS10550		GGDEF domain-containing protein	-1.69	0.03[*]
E2P69_RS14210		EAL domain-containing protein	-1.18	0.01[*]
E2P69_RS17845		EAL domain-containing protein	-0.95	0.02[*]
E2P69_RS22020		diguanylate cyclase	-0.80	-0.04[*]
E2P69_RS01315		GGDEF domain-containing protein	-0.67	-0.62
E2P69_RS10910		GGDEF domain-containing protein	-0.65	-0.28[*]

[*] Not significant

Table S6 List of differentially expressed genes in the mutant strain involved in type III secretion system.

Locus tag	Gene	Product	Log2foldchange	
			8h	16h
E2P69_RS20480		HrpB1 family type III secretion system apparatus protein	-0.41*	-1.74
E2P69_RS20475		type III secretion protein HrpB2	-0.35*	-1.53
E2P69_RS20500		type III secretion system cytoplasmic ring protein SctQ	-0.42*	-1.51
E2P69_RS20495		type III secretion system protein SctP	-0.35*	-1.49
E2P69_RS20535		CesT family type III secretion system chaperone	-0.45*	-1.49
E2P69_RS20470		type III secretion inner membrane ring lipoprotein SctJ	-0.19*	-1.48
E2P69_RS20465		type III secretion protein HrpB4	-0.15*	-1.47
E2P69_RS20505		type III secretion system export apparatus subunit SctR	-0.25*	-1.44
E2P69_RS11735	*hrpX*	helix-turn-helix transcriptional regulator	-0.14*	-1.41
E2P69_RS11740	*hrpG*	response regulator transcription factor	-0.15*	-1.39
E2P69_RS20485		type III secretion system export apparatus subunit SctU	-0.38*	-1.38
E2P69_RS20490		FHIPEP family type III secretion protein	-0.33*	-1.35
E2P69_RS20555		HpaF protein	-0.16*	-1.33
E2P69_RS20510		type III secretion system export apparatus subunit SctS	-0.30*	-1.30
E2P69_RS20460		type III secretion system stator protein SctL	-0.07*	-1.29
E2P69_RS20455		type III secretion system ATPase SctN	-0.17*	-1.27
E2P69_RS20440		type III secretion system outer membrane ring subunit SctC	-0.24*	-1.25
E2P69_RS20450		type III secretion protein HrpB7	0.01*	-1.21
E2P69_RS20445		type III secretion system export apparatus subunit SctT	-0.07*	-1.67
E2P69_RS04050		type III secretion system YopJ family effector AvrXv4	-0.11*	-0.97
E2P69_RS03165		type III PLP-dependent enzyme	0.09*	-0.93
E2P69_RS02615		type III secretion system effector protein XopK	0.04*	-0.91

* Not significant

Table S7 List of type VI secretion system genes differentially expressed in the mutant strain.

Locus tag	Gene	Product	Log2foldchange		Cluster
			8h	16h	
E2P69_RS17690		type VI secretion system contractile sheath small subunit	-0.23*	1.58	i3*
E2P69_RS22610	*hcp*	type VI secretion system tube protein	0.33*	1.46	i3*
E2P69_RS12845		thiol:disulfide interchange protein DsbA/DsbL	1.5	1.33	
E2P69_RS08700		tetratricopeptide repeat protein	0.53*	1.10	
E2P69_RS21880		type VI secretion system contractile sheath small subunit-cluster III	0.00*	1.04	i3***
E2P69_RS09395	*tssK*	type VI secretion system baseplate subunit	0.47*	1.03	i3*
E2P69_RS03325		thiol-disulfide oxidoreductase DCC family protein	0.15*	0.99	
E2P69_RS21885	*tssC*	type VI secretion system contractile sheath large subunit	0.09*	0.96	i3***
E2P69_RS09400	*tssL*	type VI secretion system protein	0.54*	0.90	i3*
E2P69_RS07570	*tagH*	type VI secretion system-associated FHA domain protein	0.25*	0.88	i3***
E2P69_RS09410	*tagF*	type VI secretion system-associated protein	1.26	0.85	i3*
E2P69_RS07575	*tssK*	type VI secretion system baseplate subunit	0.18*	0.85	i3***
E2P69_RS21910	*tssF*	type VI secretion system baseplate subunit	0.23*	0.80	i3***
E2P69_RS19505		tetratricopeptide repeat protein	0.27*	0.79	
E2P69_RS05680		RHS repeat-associated core domain-containing protein	-0.17*	0.78	
E2P69_RS21920	*clpV*	AAA family ATPase	-0.14*	0.78	i3***
E2P69_RS09415		serine/threonine-protein phosphatase	0.68	0.74	
E2P69_RS21900		type VI secretion protein, ImpE/SciE family	0.29*	0.73	i3***
E2P69_RS09365		tetratricopeptide repeat protein	-0.10*	0.73	
E2P69_RS22605		ImpE protein	-0.07*	0.72	i3*
E2P69_RS21330		carbon storage regulator CsrA	0.81	0.70	
E2P69_RS09370	*tssI*	type VI secretion system tip protein VgrG	0.09*	0.70	i3*
E2P69_RS21905	*tssE*	type VI secretion system baseplate subunit	0.09*	0.69	i3***
E2P69_RS14695		cardiolipin synthase B	0.27*	0.68	
E2P69_RS17695	*tssC*	type VI secretion system contractile sheath large subunit	0.48*	0.68	i3*
E2P69_RS09360		PAAR domain-containing	0.43*	0.67	i3*

		protein			
E2P69_RS21890	*hcp*	type VI secretion system tube protein	-0.06*	0.66	i3***
E2P69_RS09440	*tssA*	type VI secretion system protein	0.27*	0.66	i3*
E2P69_RS17420		tetratricopeptide repeat protein	0.48*	0.65	
E2P69_RS07585	*tssM*	type VI secretion system membrane subunit	0.31*	0.61	i3***
E2P69_RS07625		two-component system VirA-like sensor kinase	-0.08*	0.61	
E2P69_RS09420		protein kinase	0.20*	0.59	
E2P69_RS00975		phospholipase D family protein	0.40*	0.59	

* Not significant

Table S8 List of genes associated with T2SS, T4SS and T5SS displayed differentially expression in the *tssM* mutant strain.

Locus tag	Gene	Product	Log2foldchange	
			8h	16h
**T2SS**				
E2P69_RS00220		glycoside hydrolase family 5 protein	-1.55	-1.70
E2P69_RS00225		glycoside hydrolase family 5 protein	-0.74	-1.13
E2P69_RS00235		glycoside hydrolase family 5 protein	0.27*	-0.94
E2P69_RS19010		cellulase family glycosylhydrolase	-1.05	-0.03*
E2P69_RS02215		lipase	0.08*	-0.82
E2P69_RS15945		lipase	-0.15*	-0.78
E2P69_RS05520		xylose isomerase xylA	0.73	0.76
E2P69_RS19555		xylanase	-0.32*	0.86
E2P69_RS05380		endo-1,4-beta-xylanase	0.30*	-1.41
E2P69_RS15315		alpha-amylase family protein	0.40*	1.02
**T4SS**				
E2P69_RS21420		TrbC/VirB2 family protein	0.33*	0.65
E2P69_RS21425		VirB3 family type IV secretion system protein	0.42*	0.76
E2P69_RS21460		TrbI/VirB10 family protein	0.57*	0.95
E2P69_RS21450		type IV secretory pathway, TrbF protein	0.58	0.84
E2P69_RS21445		P-type conjugative transfer protein TrbL	0.56*	0.88
E2P69_RS14500		TrbI/VirB10 family protein	-0.96	-0.34*
E2P69_RS14505		P-type DNA transfer ATPase VirB11	-0.94	-0.04*
E2P69_RS14525		VirB4 family type IV secretion/conjugal transfer ATPase	-0.67	-0.05*
E2P69_RS19420		P-type conjugative transfer protein TrbJ	-0.94	-1.03
E2P69_RS19425		TrbI/VirB10 family protein	-0.58	-0.52*
**T5SS**				
E2P69_RS19840		autotransporter domain-containing esterase	0.95	0.47*
E2P69_RS22570		YadA-like family protein	0.25*	3.41
E2P69_RS15045		YadA-like family protein	-0.03*	-0.94
E2P69_RS17595		autotransporter-associated beta strand repeat-containing protein	-0.75	-1.36
E2P69_RS11440		DegQ family serine endoprotease	0.17*	-1.32
E2P69_RS16295		VirK family protein	-0.24*	-0.93
E2P69_RS22135		preprotein translocase subunit SecE	0.85	0.82
E2P69_RS22465		preprotein translocase subunit SecY	1.07	1.35
E2P69_RS05570		Sec-independent protein translocase subunit TatA	-0.40*	-0.68
E2P69_RS05575		twin-arginine translocase subunit TatB	-0.37*	-0.58

* Not significant

Table S9 List of differentially expressed genes coding for control of gene expression.

Locus tag	Gene	Product	Log2foldchange	
			8h	16h
**Transcription, posttranscription and translational control of gene expression**				
E2P69_RS17150	*rpoN*	RNA polymerase factor sigma-54	0.17*	0.88
E2P69_RS21330	*csrA*	carbon storage regulator CsrA	0.81	0.70
E2P69_RS10975		ribonuclease HII	0.97	1.14
E2P69_RS00065		ribonuclease P protein component	0.64	1.04
E2P69_RS10175		ribonuclease PH	-0.72	-0.20*
E2P69_RS09245		rRNA maturation RNase YbeY	-0.65	-0.56*
**Ribosomal Proteins**				
E2P69_RS18785	*rplL*	50S ribosomal protein L9	0.85	0.59
E2P69_RS10905		elongation factor Ts	0.16*	0.60
E2P69_RS22455	*rpmD*	50S ribosomal protein L30	0.76	0.61
E2P69_RS08540	*rpmJ*	50S ribosomal protein L36	0.89	0.61
E2P69_RS07105		GTPase HflX	-0.14*	0.62
E2P69_RS00070	*rpmH*	50S ribosomal protein L34	0.90	0.62
E2P69_RS22365	*rplC*	50S ribosomal protein L3	1.07	0.65
E2P69_RS22405	*rpmC*	50S ribosomal protein L29	1.19	0.68
E2P69_RS22370	*rplD*	50S ribosomal protein L4	1.04	0.74
E2P69_RS22450	*rpsE*	30S ribosomal protein S5	0.85	0.75
E2P69_RS17430		ribosome biogenesis GTPase Der	0.44*	0.79
E2P69_RS11595	*rplC*	50S ribosomal protein L19	0.71	0.81
E2P69_RS22425	*rplE*	50S ribosomal protein L5	0.70	0.82
E2P69_RS22440	*rplF*	50S ribosomal protein L6	0.75	0.83
E2P69_RS22375	*rplW*	50S ribosomal protein L23	0.93	0.84
E2P69_RS22125	*rplK*	50S ribosomal protein L11	0.90	0.85
E2P69_RS22395	*rpsC*	30S ribosomal protein S3	1.00	0.85
E2P69_RS05925	*rpmL*	50S ribosomal protein L35	0.68	0.86
E2P69_RS05930	*rplT*	50S ribosomal protein L20	0.79	0.86
E2P69_RS22120		50S ribosomal protein L1	0.83	0.90
E2P69_RS22415		50S ribosomal protein L14	0.93	0.92
E2P69_RS22115		50S ribosomal protein L10	0.95	0.92
E2P69_RS21805		50S ribosomal protein L33	1.05	0.93
E2P69_RS03460	*rpsU*	30S ribosomal protein S21	1.10	0.95
E2P69_RS22410	*rpsQ*	30S ribosomal protein S17	0.97	0.96
E2P69_RS11610	*rpsP*	30S ribosomal protein S16	0.97	0.96
E2P69_RS22390	*rplV*	50S ribosomal protein L22	0.91	0.97
E2P69_RS11810	*rpsT*	30S ribosomal protein S20	1.08	0.99

E2P69_RS11605	*rimM*	ribosome maturation factor	0.66	1.00
E2P69_RS14325	*rimP*	ribosome maturation factor	0.58	1.01
E2P69_RS22400	*rplP*	50S ribosomal protein L16	0.89	1.01
E2P69_RS22445	*rplR*	50S ribosomal protein L18	0.90	1.02
E2P69_RS22430	*rpsN*	30S ribosomal protein S14	1.09	1.04
E2P69_RS22385	*rpsS*	30S ribosomal protein S19	1.01	1.06
E2P69_RS22490	*rplQ*	50S ribosomal protein L17	1.09	1.06
E2P69_RS11820	*rpmA*	50S ribosomal protein L27	0.88	1.06
E2P69_RS21800	*rpmB*	50S ribosomal protein L28	1.03	1.09
E2P69_RS22380	*rplB*	50S ribosomal protein L2	1.16	1.11
E2P69_RS22420	*rplX*	50S ribosomal protein L24	0.92	1.12
E2P69_RS22460	*rplO*	50S ribosomal protein L15	0.88	1.13
E2P69_RS22110	*rplL*	50S ribosomal protein L7/L12	0.99	1.13
E2P69_RS22085		elongation factor G	1.00	1.18
E2P69_RS11825	*rplU*	50S ribosomal protein L21	0.91	1.19
E2P69_RS14340		30S ribosome-binding factor RbfA	0.99	1.19
E2P69_RS22345		elongation factor Tu	1.15	1.48
E2P69_RS11815		GTPase ObgE	1.68	2.41
E2P69_RS07135		ribosome assembly RNA-binding protein YhbY	-0.78	-0.51*
E2P69_RS04970		6-carboxytetrahydropterin synthase QueD	1.17	0.53*
E2P69_RS11040		queuosine precursor transporter	0.35*	-0.67
E2P69_RS14330		transcription termination/ antitermination protein NusA	0.46*	1.06
E2P69_RS22130		transcription termination/ antitermination protein NusG	1.02	1.12

* Not significant

Table S10 Differentially expressed cell wall biogenesis and cell division genes in the mutant.

Locus tag	Gene	Product	Log2foldchange	
			8h	16h
E2P69_RS06005		glycosyltransferase	0.624	0.91
E2P69_RS19735		glycosyltransferase family 2 protein	-0.36*	0.92
E2P69_RS06015		WecB/TagA/CpsF family glycosyltransferase	0.24*	0.95
E2P69_RS05035		glycoside hydrolase family 99-like domain-containing protein	0.37*	1.00
E2P69_RS19740		glycosyltransferase	0.17*	1.27
E2P69_RS05995		glycosyltransferase	0.57*	0.81
E2P69_RS05990		glycosyltransferase family 4 protein	0.41*	0.81
E2P69_RS17085		glycosyltransferase	-0.39*	0.84
E2P69_RS07810		glycosyltransferase	0.38*	0.88
E2P69_RS10970		lipid-A-disaccharide synthase	0.84	1.10
E2P69_RS03820		O-antigen ligase family protein	0.77	0.62
E2P69_RS05050		GtrA family protein	-0.37*	0.76
E2P69_RS22825		mannose-1-phosphate guanylyltransferase/mannose-6-phosphate isomerase	-0.07*	0.79
E2P69_RS08060		tetraacyldisaccharide 4'-kinase LpxK	0.03*	0.59
E2P69_RS08055		3-deoxy-manno-octulosonate cytidylyltransferase	0.11*	1.12
E2P69_RS05980		acyltransferase family protein	0.63	0.75
E2P69_RS06020		gum cluster cupin domain	0.64	0.33*
E2P69_RS05965		gumC family protein	0.64	0.17*
E2P69_RS17100		UDP-3-O-(3-hydroxymyristoyl)glucosamine N-acyltransferase	-0.61	0.88
E2P69_RS10955		UDP-3-O-(3-hydroxymyristoyl)glucosamine N-acyltransferase	0.88	0.92
E2P69_RS18425		UDP-N-acetylmuramoyl-L-alanine--D-glutamate ligase	0.40*	1.06
E2P69_RS10965		acyl-ACP--UDP-N-acetylglucosamine O-acyltransferase	1.08	1.12
E2P69_RS17090		polysaccharide pyruvyl transferase family protein	-0.53*	1.39
E2P69_RS11805		murein biosynthesis integral membrane protein MurJ	1.09	1.68
E2P69_RS14115		transglycosylase SLT domain-containing protein	0.62	0.72
E2P69_RS14605		lytic murein transglycosylase MltB	0.31*	0.72

E2P69_RS14510		lytic transglycosylase domain-containing protein	-0.67	-0.29*
E2P69_RS20410		lytic transglycosylase domain-containing protein MltE	-0.14*	-1.42
E2P69_RS13385		FtsX-like permease family protein	0.42*	0.79
E2P69_RS12520		rod shape-determining protein MreC	0.99	1.01
E2P69_RS12525		rod shape-determining protein MreD	0.76	0.98
E2P69_RS12535		rod shape-determining protein RodA	0.01*	0.60
E2P69_RS17350		DNA translocase FtsK	0.45*	0.70
E2P69_RS13080		cell division protein FtsL	0.22*	-0.69
E2P69_RS13125		cell division protein FtsQ/DivIB	0.10*	-0.67
E2P69_RS18205		cell envelope integrity protein CreD	0.21*	-0.99

* Not significant

Table S11 List of genes associated with inorganic ion transport and metabolism.

Locus tag	Gene	Product	Log2foldchange	
			8h	16h
E2P69_RS17150	*rpoN*	RNA polymerase factor sigma-54	0.17*	0.88
E2P69_RS17540		NarK/NasA family nitrate transporter	-0.01*	1.42
E2P69_RS17550	*nirD*	nitrite reductase small subunit	-0.15*	1.60
E2P69_RS17545	*nirB*	NAD(P)/FAD-dependent oxidoreductase	-0.14*	1.61
E2P69_RS11645		response regulator NtrC family	-0.21*	0.83
E2P69_RS01055		nitrogen regulation protein NR(I) NtrC	-0.12*	1.36
E2P69_RS01040		P-II family nitrogen regulator	-0.08*	1.07
E2P69_RS15545		P-II family nitrogen regulator	0.35*	0.58
E2P69_RS01045		ammonium transporter	0.06*	1.37
E2P69_RS16795	*phoR*	phosphate regulon sensor histidine kinase	0.43*	0.67
E2P69_RS06675		PhoH family protein	0.37*	0.64
E2P69_RS18535	*pstS*	phosphate ABC transporter substrate-binding protein	0.20*	0.81
E2P69_RS18520	*pstB*	phosphate ABC transporter ATP-binding protein	0.02*	0.94
E2P69_RS18515	*phoU*	phosphate signaling complex protein	-0.05*	1.03
E2P69_RS18525	*pstA*	phosphate ABC transporter permease	0.12*	1.09
E2P69_RS18530	*pstC*	phosphate ABC transporter permease subunit	0.21*	1.14
E2P69_RS20335		histidine-type phosphatase	0.58	1.28
E2P69_RS18060		alkaline phosphatase family protein	-0.20*	0.90
E2P69_RS16410		glycerophosphodiester phosphodiesterase family protein	0.43*	0.99
E2P69_RS15410	*oprP*	porin	-1.00	0.21*
E2P69_RS02800	*pqqB*	pyrroloquinoline quinone biosynthesis protein	-0.11*	-0.78
E2P69_RS02810	*pqqD*	pyrroloquinoline quinone biosynthesis peptide chaperone	-0.19*	-0.69
E2P69_RS02805	*pqqC*	pyrroloquinoline-quinone synthase	0.03*	-0.67
E2P69_RS02815	*pqqE*	pyrroloquinoline quinone biosynthesis protein	0.07*	-0.63
E2P69_RS07775		YeeE/YedE family protein	0.59	2.13
E2P69_RS12595		sulfite exporter TauE/SafE family protein	1.22	1.38

E2P69_RS07790		sulfite exporter TauE/SafE family protein	0.67	0.71
E2P69_RS13380		TauD/TfdA family dioxygenase	-0.16	-1.08
E2P69_RS07925		sulfurtransferase	-0.54	-0.62
E2P69_RS20745		cysteine synthase A	-0.14*	-0.74
E2P69_RS20680	*cysD*	sulfate adenylyltransferase subunit CysD	-0.09*	-0.61
E2P69_RS21230		ferrous iron transport protein A	-0.11*	-0.70
E2P69_RS10085		rubredoxin	-0.47*	-0.96
E2P69_RS17575		ferritin-like domain-containing protein	-1.03	0.05*
E2P69_RS20330		siderophore-interacting protein	0.62	0.67
E2P69_RS00875		TonB-dependent siderophore receptor	0.64	0.11*

* Not significant

Table S12 List of dysregulated genes involved in amino acid and energy production.

Locus tag	Gene	Product	Log2foldchange	
			8h	16h
E2P69_RS21115	*hisB*	bifunctional histidinol-phosphatase/imidazoleglycerol-phosphate dehydratase	-0.13*	1.17
E2P69_RS21130	*hisF*	imidazole glycerol phosphate synthase subunit	-0.21*	1.20
E2P69_RS21110	*hisC*	histidinol-phosphate transaminase	-0.10*	1.21
E2P69_RS21120	*hisH*	imidazole glycerol phosphate synthase subunit	0.02*	1.24
E2P69_RS21135	*hisIE*	bifunctional phosphoribosyl-AMP cyclohydrolase/phosphoribosyl-ATP diphosphatase	-0.57*	1.27
E2P69_RS21105	*hisD*	histidinol dehydrogenase	-0.28*	1.28
E2P69_RS21125	*hisA*	1-(5-phosphoribosyl)-5-[(5-phosphoribosylamino) methylideneamino] imidazole-4-carboxamide isomerase	0.19*	1.59
E2P69_RS21100	*hisG*	ATP phosphoribosyltransferase	-0.09*	1.62
E2P69_RS21095		trp operon repressor	-0.28*	1.75
E2P69_RS21025	*carB*	carbamoyl-phosphate synthase large subunit	0.45*	0.74
E2P69_RS08780	*argC*	N-acetyl-gamma-glutamyl-phosphate reductase	0.46*	1.17
E2P69_RS08795	*argE*	acetylornithine deacetylase	0.24*	1.24
E2P69_RS08775	*argH*	argininosuccinate lyase	0.42*	1.24
E2P69_RS08790	*argB*	acetylglutamate kinase	0.23*	1.28
E2P69_RS07515	*aroA*	3-phosphoshikimate 1-carboxyvinyltransferase	0.64	0.97
E2P69_RS19925		3-deoxy-7-phosphoheptulonate synthase	0.63	0.72
E2P69_RS16500		aminodeoxychorismate synthase component I	0.82	0.62
E2P69_RS04815		chorismate mutase	0.75	-0.19*
E2P69_RS09875		3-isopropylmalate dehydrogenase	-0.48*	-0.64
E2P69_RS09870		3-isopropylmalate dehydratase small subunit	-0.53*	-0.97
E2P69_RS09865		3-isopropylmalate dehydratase large subunit	-0.60	-0.80

E2P69_RS08130		succinate dehydrogenase, cytochrome b556 subunit	-0.72	-0.97
E2P69_RS08125		succinate dehydrogenase, hydrophobic membrane anchor protein	-0.69	-0.93
E2P69_RS08120		succinate dehydrogenase flavoprotein subunit	-0.61	-0.75
E2P69_RS08110		succinate dehydrogenase iron-sulfur subunit	-0.71	-0.65
E2P69_RS08725		cytochrome ubiquinol oxidase subunit I	-0.79	-0.86
E2P69_RS08195		c-type cytochrome	-0.69	-0.84
E2P69_RS13540		protocatechuate 3,4-dioxygenase subunit alpha	-0.33[*]	-1.41
E2P69_RS21585		protocatechuate 3,4-dioxygenase subunit beta	0.18[*]	-0.79

[*] Not significant

Table S13 Stress response genes differentially expressed in the mutant strain.

Locus tag	Gene	Product	Log2foldchange	
			8h	16h
E2P69_RS16345		1,4-alpha-glucan branching enzyme	0.14*	0.72
E2P69_RS16335		4-alpha-glucanotransferase	-0.07*	0.73
E2P69_RS16330	*treY*	malto-oligosyltrehalose synthase	-0.02*	0.60
E2P69_RS16340	*treZ*	malto-oligosyltrehalose trehalohydrolase	-0.03*	0.69
E2P69_RS12250	*treA*	alpha,alpha-trehalase	-0.78	-0.77
E2P69_RS08795		acetylornithine deacetylase	0.24*	1.24
E2P69_RS12820		betaine-aldehyde dehydrogenase	0.43*	1.52
E2P69_RS15510	*speA*	arginine decarboxylase	-0.25*	0.66
E2P69_RS12825	*betT*	choline BCCT transporter	0.70	1.38
E2P69_RS08555		agmatine deiminase family protein	-0.00*	0.62
E2P69_RS09280	*potB*	ABC transporter permease subunit	0.41*	0.85
E2P69_RS09275	*potC*	ABC transporter permease subunit	0.21*	1.34
E2P69_RS06565	*potA*	polyamine ABC transporter ATP-binding protein	0.10*	0.89
E2P69_RS15800		CsbD family protein	1.15	0.09*
E2P69_RS14465	*uvrB*	excinuclease ABC subunit	0.66	0.73
E2P69_RS11830	*uvrA*	excinuclease ABC subunit	0.39*	0.58
E2P69_RS10375		OmpW family protein	-0.48*	-0.83
E2P69_RS01815		OsmC family protein	-0.09*	-0.60
E2P69_RS15710	*rpoE*	RNA polymerase sigma factor	1.05	1.48
E2P69_RS09345	*rpoE*	RNA polymerase sigma factor	-0.14*	0.83
E2P69_RS11450	*rpoE*	RNA polymerase sigma factor	0.29*	-1.00

* Not significant
